# Supplementary figures and images for: Researching COVID to Enhance Recovery (RECOVER) adult study protocol: Rationale, objectives, and design
Source: PLoS One. 2023 Jun 23;18(6):e0286297. doi: 10.1371/journal.pone.0286297 (PMC10289397; doi:10.1371/journal.pone.0286297)

**S1 Figure: Protocol Development Timeline**

**
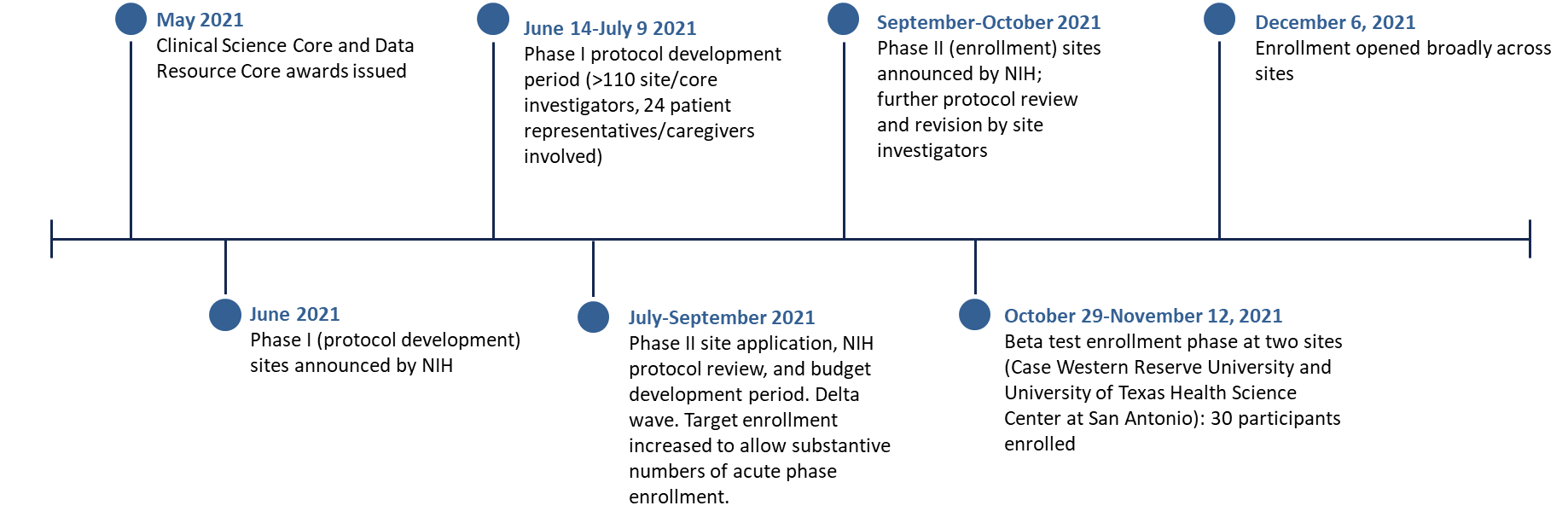
**

Supplement: S1 Fig — (DOCX) [file pone.0286297.s001.docx]
