# Supplementary material for: Researching COVID to Enhance Recovery (RECOVER) adult study protocol: Rationale, objectives, and design
Source: PLoS One. 2023 Jun 23;18(6):e0286297. doi: 10.1371/journal.pone.0286297 (PMC10289397; doi:10.1371/journal.pone.0286297)
Supplement: S2 Fig — (DOCX) [file pone.0286297.s002.docx]

**S2 Figure: RECOVER Consortium Oversight Structure**


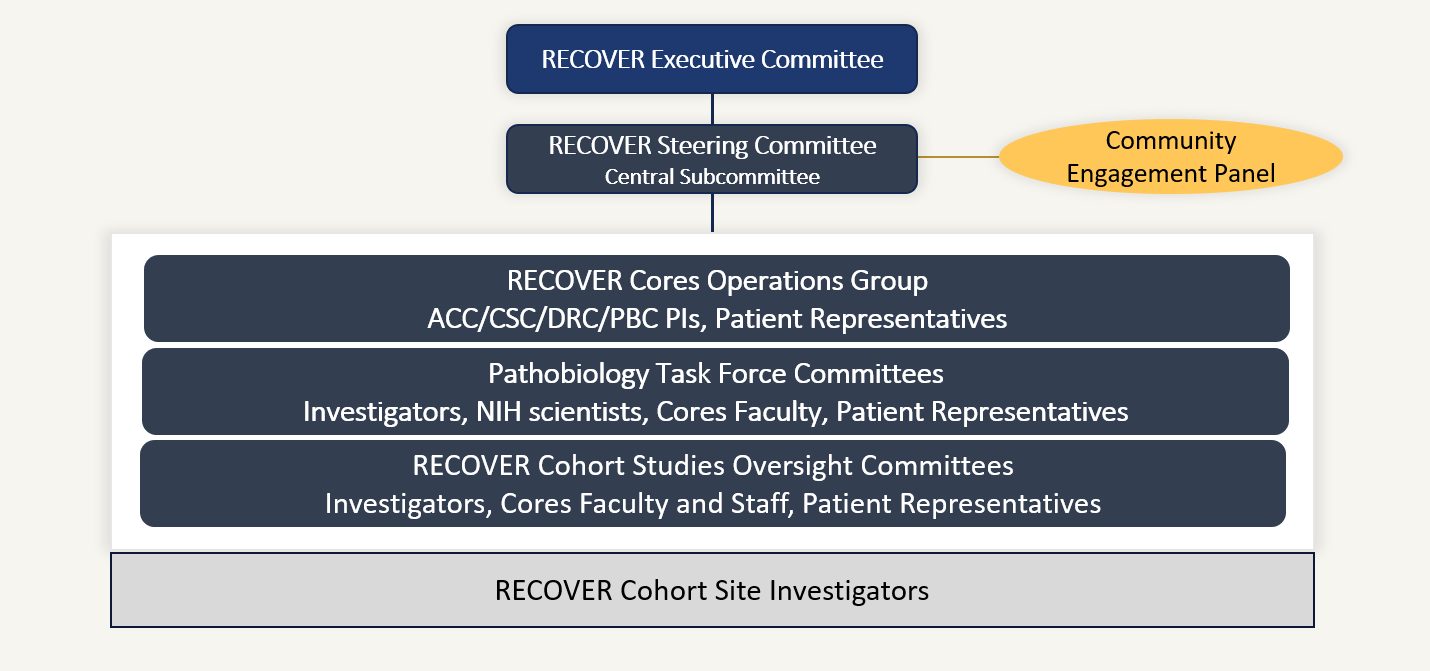


ACC: Administrative Coordinating Center; CSC: Clinical Science Core; DRC: Data Resource Core; PBC: PASC Biorepository Core; PI: Principal Investigator; NIH: National Institutes of Health
