# Supplementary material for: Researching COVID to Enhance Recovery (RECOVER) adult study protocol: Rationale, objectives, and design
Source: PLoS One. 2023 Jun 23;18(6):e0286297. doi: 10.1371/journal.pone.0286297 (PMC10289397; doi:10.1371/journal.pone.0286297)
Supplement: S1 Table — (DOCX) [file pone.0286297.s003.docx]

**S1 Table: Hubs and Enrolling Sites**

| **Hub/consortium name** | **Enrolling site** | **Location** |
| --- | --- | --- |
| Atlanta RECOVER Clinical Research Site (CRS) | Atlanta VA Health Care System | Georgia |
| Atlanta RECOVER Clinical Research Site (CRS) | Emory Healthcare, Hope Clinic | Georgia |
| Atlanta RECOVER Clinical Research Site (CRS) | Grady Health System | Georgia |
| Atlanta RECOVER Clinical Research Site (CRS) | Kaiser Permanente of Georgia | Georgia |
| Atlanta RECOVER Clinical Research Site (CRS) | Morehouse School of Medicine | Georgia |
| Boston COVID-19 Recovery Cohort | Beth Israel Lahey Health | Massachusetts |
| Boston COVID-19 Recovery Cohort | Boston University, Boston Medical Center | Massachusetts |
| Boston COVID-19 Recovery Cohort | Brigham and Women's Hospital | Massachusetts |
| Boston COVID-19 Recovery Cohort | Cambridge Health Alliance | Massachusetts |
| Boston COVID-19 Recovery Cohort | Massachusetts General Hospital | Massachusetts |
| Boston COVID-19 Recovery Cohort | South Shore Hospital | Massachusetts |
| Boston COVID-19 Recovery Cohort | Tufts Medical Center | Massachusetts |
| Deep South SARS-CoV-2 Recovery Cohort | University Medical Center New Orleans | Louisiana |
| Deep South SARS-CoV-2 Recovery Cohort | University of Alabama at Birmingham | Alabama |
| Deep South SARS-CoV-2 Recovery Cohort | University of South Alabama | Alabama |
| Howard University | Howard University | Washington DC |
| Howard University | Mercy Medical Center, University of Maryland | Maryland |
| IDeA States Consortium for Clinical Research (ISCORE) | Hispanic Alliance for Clinical and Translational Research, University of Puerto Rico Medical Science Campus | Puerto Rico |
| IDeA States Consortium for Clinical Research (ISCORE) | Louisiana State University Health Sciences Center New Orleans | Louisiana |
| IDeA States Consortium for Clinical Research (ISCORE) | Pennington Biomedical Research Center | Louisiana |
| IDeA States Consortium for Clinical Research (ISCORE) | MaineHealth Northern New England IDeA CTR | Maine |
| IDeA States Consortium for Clinical Research (ISCORE) | Sanford Health | South Dakota |
| IDeA States Consortium for Clinical Research (ISCORE) | Tulane School of Medicine | Louisiana |
| IDeA States Consortium for Clinical Research (ISCORE) | University of Hawaii Mountain West CTR | Hawaii |
| IDeA States Consortium for Clinical Research (ISCORE) | University of Kansas Medical Center | Kansas |
| IDeA States Consortium for Clinical Research (ISCORE) | University of Kentucky | Kentucky |
| IDeA States Consortium for Clinical Research (ISCORE) | University of Mississippi Medical Center | Mississippi |
| IDeA States Consortium for Clinical Research (ISCORE) | University of Nebraska Medical Center | Nebraska |
| IDeA States Consortium for Clinical Research (ISCORE) | University of Oklahoma Health Sciences Center | Oklahoma |
| IDeA States Consortium for Clinical Research (ISCORE) | West Virginia University | West Virginia |
| ILLInet | BrightStar Community Outreach | Illinois |
| ILLInet | Illinois Unidos | Illinois |
| ILLInet | Mile Square Health Centers | Illinois |
| ILLInet | Peoria City, County Health Department | Illinois |
| ILLInet | UI Hospital & Clinics | Illinois |
| Mount Sinai PASC Coalition (SinaiPACT) | Icahn School of Medicine at Moloraunt Sinai | New York |
| Mountain States PASC Collaborative | Bateman Horne Center | Utah |
| Mountain States PASC Collaborative | Denver Health and Hospital Authority | Colorado |
| Mountain States PASC Collaborative | Intermountain Healthcare | Utah |
| Mountain States PASC Collaborative | University of Colorado Anschutz Medical Campus | Colorado |
| Mountain States PASC Collaborative | University of New Mexico Health Sciences Center | New Mexico |
| Mountain States PASC Collaborative | University of Utah | Utah |
| NorthEast Ohio Covid United for REcovery (NEO-CURE) | Case Western Reserve University, University Hospitals Health System | Ohio |
| NorthEast Ohio Covid United for REcovery (NEO-CURE) | Case Western Reserve University, The MetroHealth System | Ohio |
| The Pacific Northwest Consortium for Post-Acute Sequelae of SARS-CoV-2 Infection | Institute for Systems Biology | Washington |
| The Pacific Northwest Consortium for Post-Acute Sequelae of SARS-CoV-2 Infection | Providence Swedish Medical Center | Washington |
| The Pacific Northwest Consortium for Post-Acute Sequelae of SARS-CoV-2 Infection | University of Washington | Washington |
| The Pacific Northwest Consortium for Post-Acute Sequelae of SARS-CoV-2 Infection | Providence Sacred Heart Medical Center | Washington |
| The Pacific Northwest Consortium for Post-Acute Sequelae of SARS-CoV-2 Infection | Providence Regional Medical Center Everett | Washington |
| The Pacific Northwest Consortium for Post-Acute Sequelae of SARS-CoV-2 Infection | Cedars Sinai LA | California |
| PREVAIL South Texas | University of Texas Health Science Center at San Antonio | Texas |
| PREVAIL South Texas | University of Texas Education and Research Center at Laredo | Texas |
| Stanford Post-Acute Recovery Cohort (SPARC) | Stanford University | California |
| Stanford Post-Acute Recovery Cohort (SPARC) | Stanford Health Care Tri-Valley | California |
| United Against COVID - AZPC3 Consortium | Banner University Medical Center Tucson | Arizona |
| United Against COVID - AZPC3 Consortium | Banner University Medical Center, Phoenix | Arizona |
| United Against COVID - AZPC3 Consortium | University of Arizona | Arizona |
| University of California San Francisco | Chan Zuckerberg San Francisco General Hospital | California |
| University of California San Francisco | San Mateo County Health Department | California |
| University of California San Francisco | UCSF Parnassus Medical Center | California |
| PRIORITY: Post-Acute Sequelae of SARS-CoV-2 in Pregnant Women and their Children | University of California San Francisco, and nationally through home visits | California |
| MFMU PASC-PREG | University of Utah (Pregnancy) | Utah |
| MFMU PASC-PREG | University of Alabama at Birmingham (pregnancy) | Alabama |
| MFMU PASC-PREG | University of Texas Health Science Center at Houston | Texas |
| MFMU PASC-PREG | University of Texas Medical Branch at Galveston | Texas |
| MFMU PASC-PREG | UH MacDonald's Women's Hospital | Ohio |
| MFMU PASC-PREG | Yale University | Connecticut |
| MFMU PASC-PREG | University of Pittsburgh, Magee | Pennsylvania |
| MFMU PASC-PREG | University of Pennsylvania | Pennsylvania |
| MFMU PASC-PREG | University of North Carolina Chapel Hill | North Carolina |
| MFMU PASC-PREG | Saint Peter's University Hospital | New Jersey |
| MFMU PASC-PREG | Ohio State University | Ohio |
| MFMU PASC-PREG | Northwestern University | Illinois |
| MFMU PASC-PREG | NorthShore University HealthSystem | Illinois |
| MFMU PASC-PREG | New York-Presbyterian, Queens | New York |
| MFMU PASC-PREG | Miami Valley Hospital | Ohio |
| MFMU PASC-PREG | Case Western, MetroHealth Medical | Ohio |
| MFMU PASC-PREG | Medical College of Wisconsin | Wisconsin |
| MFMU PASC-PREG | Good Samaritan | Ohio |
| MFMU PASC-PREG | Duke University Medical Center | North Carolina |
| MFMU PASC-PREG | ChristianaCare | Delaware |
| MFMU PASC-PREG | Memorial City Medical Center | Texas |
| MFMU PASC-PREG | Brown University, Women & Infants Hospital of Rhode Island | Rhode Island |
| MFMU PASC-PREG | LBJ Hospital | Texas |
| MFMU PASC-PREG | Columbia University | New York |
| MFMU PASC-PREG | University of Colorado | Colorado |
