# Supplementary material for: Researching COVID to Enhance Recovery (RECOVER) adult study protocol: Rationale, objectives, and design
Source: PLoS One. 2023 Jun 23;18(6):e0286297. doi: 10.1371/journal.pone.0286297 (PMC10289397; doi:10.1371/journal.pone.0286297)
Supplement: S2 Table — (DOCX) [file pone.0286297.s004.docx]

**S2 Table: Survey Topics as of Protocol Version 7.0***

| **Survey instrument** | **Topic** | **Asked at follow up** | **Source of question, if not RECOVER** |
| --- | --- | --- | --- |
| Demographics | Name and contact information (retained locally) | ✓ |  |
| Demographics | Alternate contacts (retained locally) | ✓ |  |
| Demographics | Date of birth |  |  |
| Demographics | Race and ethnicity |  | All of Us |
| Demographics | Biological sex |  | RADx Global Codebook |
| Demographics | Gender identity |  | All of Us |
| Demographics | Sexual orientation |  | All of Us |
| Social determinants | Education |  | NHANES |
| Social determinants | Number of people in household |  | American Community Survey |
| Social determinants | Homelessness | ✓ |  |
| Social determinants | Description of living place |  | American Community Survey |
| Demographics | Marital status | ✓ | BRFSS |
| Social determinants | Employment | ✓ | RADx Global Codebook |
| Social determinants | Health insurance | ✓ | American Community Survey |
| Social determinants | Birthplace |  | American Community Survey |
| Social determinants | Primary language |  | California Health Interview Survey |
| Social determinants | Fluency in English |  | California Health Interview Survey |
| Social determinants | Income in 2019 |  | National Health Interview Survey |
| Social determinants | Financial insecurity | ✓ | RAND American Life Panel Impacts of COVID-19 Survey |
| Social determinants | Food insecurity |  | Hunger Vital Sign |
| Social determinants | Access to health care |  | National Health Interview Survey |
| Social determinants | Social support |  | Medical Outcomes Study (MOS) Social Support Survey |
| ~~Social determinants~~ | ~~Loss of insurance because of COVID pandemic~~ |  |  |
| Social determinants | Community cohesion |  | Project on Human Development in Chicago Neighborhoods |
| Social determinants | Discrimination |  | Everyday Discrimination Scale |
| Social determinants | Alcohol and substance use | ✓ | TAPS Part 1 |
| Baseline disability | Baseline disability |  | CDC Disability |
| Acute COVID | Diagnosis method |  |  |
| Acute COVID | Site and level of care for initial infection |  | WHO PASC CRF |
| Acute COVID | Treatments received for initial infection |  | Modified WHO PASC CRF |
| Pregnancy | Pregnancy status | ✓ | RADx-UP |
| Pregnancy | Pregnancy outcomes | ✓ | Modified WHO PASC CRF |
| Vaccination | Vaccination status and vaccine details | ✓ | WHO PASC CRF |
| Comorbidity | Immunocompromised condition and specific types | ✓ |  |
| Comorbidity | Rheumatologic, autoimmune or connective tissue disease and specific types | ✓ |  |
| Comorbidity | Diabetes and specific type | ✓ |  |
| Comorbidity | Kidney disease and specific type | ✓ |  |
| Comorbidity | Active cancer and specific type | ✓ |  |
| Comorbidity | Dementia or cognitive impairment and specific type | ✓ | NIH/NINDS NeuroCOVID Databank |
| Comorbidity | Central nervous system infection, inflammatory disease or demyelinating disease and specific type | ✓ | NIH/NINDS NeuroCOVID Databank |
| Comorbidity | Seizure disorder | ✓ |  |
| Comorbidity | Neuromuscular disease and specific type | ✓ | NIH/NINDS NeuroCOVID Databank |
| Comorbidity | Movement disorder and specific type | ✓ | NIH/NINDS NeuroCOVID Databank |
| Comorbidity | Cardiovascular disease and specific type | ✓ |  |
| Comorbidity | Stroke or bleed and specific type | ✓ | NIH/NINDS NeuroCOVID Databank |
| Comorbidity | Asthma | ✓ |  |
| Comorbidity | Chronic obstructive pulmonary disease | ✓ |  |
| Comorbidity | Other chronic lung disease | ✓ |  |
| Comorbidity | Use of oxygen at home | ✓ |  |
| Comorbidity | Anxiety, depression or PTSD | ✓ |  |
| Comorbidity | Schizophrenia or bipolar disorder | ✓ |  |
| Comorbidity | Other mental health disorder | ✓ |  |
| Comorbidity | Chronic liver disease | ✓ |  |
| Comorbidity | Sickle cell anemia | ✓ |  |
| Comorbidity | Chronic pain syndrome or fibromyalgia | ✓ |  |
| Comorbidity | Myalgic encephalomyelitis/chronic fatigue syndrome | ✓ |  |
| Comorbidity | POTS or other form of dysautonomia or autonomic dysfunction and specific type | ✓ |  |
| Comorbidity | Obesity | ✓ |  |
| Comorbidity | Polycystic ovarian syndrome | ✓ |  |
| Medications | Complete medication list | ✓ |  |
| Symptoms | Global health | ✓ | PROMIS-10 v1.2 |
| Symptoms | Quality of life | ✓ | PROMIS-10 v1.2 |
| Symptoms | Physical health and function, and details | ✓ | PROMIS10 v1.2, PROMIS physical function SF 4a |
| Symptoms | Mental health and thinking | ✓ | PROMIS-10 v1.2 |
| Symptoms | Social activities satisfaction and ability | ✓ | PROMIS-10 v1.2 |
| Symptoms | Anxiety, depression, irritability | ✓ | PROMIS10 v1.2 |
| Symptoms | Fatigue | ✓ | PROMIS 10 v1.2 |
| Symptoms | Pain | ✓ | PROMIS 10 v1.2 |
| Symptoms | Post-exertional malaise | ✓ |  |
| Symptoms | Weakness in limbs | ✓ | WHO PASC CRF |
| Symptoms | Fever, chills, sweats or flushing | ✓ |  |
| Symptoms | Loss of or change in smell or taste | ✓ |  |
| Symptoms | Pain and details | ✓ |  |
| Symptoms | Headache and details | ✓ | Headache Inventory Test-6 |
| Symptoms | Chest pain and details | ✓ | Seattle Angina Questionnaire |
| Symptoms | Shortness of breath or trouble breathing and details | ✓ | modified Medical Research Council scale |
| Symptoms | Cough | ✓ |  |
| Symptoms | Palpitations, racing heart, arrhythmia, skipped beats | ✓ |  |
| Symptoms | Swelling of lower legs and details | ✓ |  |
| Symptoms | Gastrointestinal symptoms and details | ✓ | COMPASS-31 |
| Symptoms | Bladder problems and details | ✓ | COMPASS-31 |
| Symptoms | Nerve problems and details | ✓ | PROMIS physical function 4a;  Neuro-QoL SF Upper extremity function v1.0; Michigan neuropathy screener |
| Symptoms | Problems thinking or concentrating and details | ✓ | Neuro-QoL Cognitive Function SF v2.0 |
| Symptoms | Problems with sleep and details | ✓ | PROMIS Sleep Disturbance 8a |
| Symptoms | Orthostatic symptoms and details | ✓ | COMPASS-31 |
| Symptoms | Skin color changes and details | ✓ | COMPASS-31 |
| Symptoms | Skin rash | ✓ |  |
| Symptoms | Changes in sweating | ✓ | COMPASS-31 |
| Symptoms | Excessively dry eyes | ✓ | COMPASS-31 |
| Symptoms | Excessively dry mouth | ✓ | COMPASS-31 |
| Symptoms | Excessive thirst | ✓ |  |
| Symptoms | Vision problems (blurry, light sensitivity, difficulty reading or focusing, floaters, flashing lights, "snow") and details | ✓ | COMPASS-31, NEI Visual Functioning Questionnaire 25 |
| Symptoms | Problems with hearing (hearing loss, ringing in ears) and details | ✓ |  |
| Symptoms | Hair loss | ✓ |  |
| Symptoms | Problems with teeth or gums | ✓ |  |
| Symptoms | Change in menstruation or menopause and details | ✓ |  |
| Symptoms | Changes in desire for, comfort with or capacity for sex | ✓ | SHOW-Q (females), UCLA Prostate Cancer Index sexual function (males) |
| Symptoms | Depression screen and assessment | ✓ | PHQ-8 |
| Symptoms | Suicidality screen and assessment | ✓ | ~~PHQ-9,~~ CSSRS suicidality screener |
| Symptoms | Anxiety screen and assessment | ✓ | Generalized Anxiety Disorder-7 |
| Symptoms | Prolonged grief screen and assessment | ✓ | Prolonged Grief-13r |
| Symptoms | Stress | ✓ | Perceived Stress Scale-4 |
| Post-COVID utilization | Hospitalization since COVID or last assessment | ✓ |  |
| Post-COVID utilization | Emergency department visit since COVID or last assessment | ✓ |  |

*Strikethrough text: Measure in earlier version of the protocol
