# Supplementary material for: Researching COVID to Enhance Recovery (RECOVER) adult study protocol: Rationale, objectives, and design
Source: PLoS One. 2023 Jun 23;18(6):e0286297. doi: 10.1371/journal.pone.0286297 (PMC10289397; doi:10.1371/journal.pone.0286297)
Supplement: S3 Table — (DOCX) [file pone.0286297.s005.docx]

**S3 Table: Tier 1 Assessments**

| **Category** | **Assessment** | **In original protocol Tier 1** | **In current protocol Tier 1 (v7.0)** |
| --- | --- | --- | --- |
| Clinical assessment | Height, weight, body mass index | ✓ | ✓ |
| Clinical assessment | Waist circumference | ✓ | ✓ |
| Clinical assessment | Seated vital signs (blood pressure, heart rate, respiratory rate, oxygen saturation) | ✓ | ✓ |
| Clinical assessment | 30 second sit to stand | ✓ | ✓ |
| Clinical assessment | Active standing test | ✓ | ✓ |
| Clinical assessment | Wearable with continuous remote monitoring for electrocardiogram, respiratory rate, oxygenation, sleep fragmentation, actigraphy | ✓ | ✓ |
| Laboratory study | Comprehensive metabolic panel with cystatin-C | ✓ | ✓ |
| Laboratory study | Complete blood count with differential | ✓ | ✓ |
| Laboratory study | Lipid panel | ✓ | ✓ |
| Laboratory study | Hemoglobin A1c | ✓ | ✓ |
| Laboratory study | Prothrombin time, international normalized ratio, partial thromboplastin time |  | ✓ |
| Laboratory study | D-dimer |  | ✓ |
| Laboratory study | Troponin |  | ✓ |
| Laboratory study | N-terminal pro-brain natriuretic peptide |  | ✓ |
| Laboratory study | Thyroid stimulating hormone, free T4 | ✓ | ✓ |
| Laboratory study | Anti nuclear antibody | ✓ |  |
| Laboratory study | Rheumatoid factor | ✓ |  |
| Laboratory study | Anti-cyclic citrullinated peptide antibodies | ✓ |  |
| Laboratory study | EBV anti early antigen IgG, viral capsid IgM, viral capsid IgG, nuclear antigen IgG | ✓ |  |
| Laboratory study | 25-hydroxy vitamin D | ✓ | ✓ |
| Laboratory study | Urinalysis | ✓ | ✓ |
| Laboratory study | Urine microalbumin and creatinine | ✓ | ✓ |
| Laboratory study | hsCRP | ✓ | ✓ |
| Laboratory study | SARS-CoV-2 spike and/or nucleocapsid antibody | ✓ | ✓ |
| Laboratory study | SARS-CoV-2 NAAT | ✓ | ✓ |
