# Supplementary material for: Researching COVID to Enhance Recovery (RECOVER) adult study protocol: Rationale, objectives, and design
Source: PLoS One. 2023 Jun 23;18(6):e0286297. doi: 10.1371/journal.pone.0286297 (PMC10289397; doi:10.1371/journal.pone.0286297)
Supplement: S4 Table — (DOCX) [file pone.0286297.s006.docx]

**S4 Table: Tier 2 Assessments**

| **Category** | **Assessment** | **In original protocol Tier 2** | **In current protocol Tier 2 (v7.0)** | **Performed in pregnant, <3 month post-partum** |
| --- | --- | --- | --- | --- |
| Examination | Home sleep test | ✓ | ✓ | ✓ |
| Examination | 6 minute walk test | ✓ | ✓ | ✓ |
| Examination | Complete neurologic exam | ✓ |  | ✓ |
| Examination | Neuropathy exam |  | ✓ |  |
| Examination | Cardiovagal innervation testing | ✓ |  | ✓ |
| Examination | Rehabilitation exam | ✓ | ✓ | ✓ |
| Examination | Ears, nose, throat exam |  | ✓ | ✓ |
| Examination | Mini International Neuropsychiatric Interview (MINI) | ✓ | ✓ | ✓ |
| Examination | Vision screen | ✓ | ✓ | ✓ |
| Examination | Smell Test | ✓ | ✓ | ✓ |
| Examination | NIH Toolbox oral reading recognition test age 3+ v2.0 | ✓ | ✓ | ✓ |
| Examination | NIH Toolbox picture vocabulary test age 3+ v2.0 | ✓ | ✓ | ✓ |
| Examination | NIH Toolbox auditory verbal learning test (Rey) 8+ v2.0 | ✓ | ✓ | ✓ |
| Examination | NIH Flanker inhibitory control and attention test age 12+ v2.1 | ✓ | ✓ | ✓ |
| Examination | NIH Toolbox pattern comparison processing speed test age 7+ v2.1 | ✓ | ✓ | ✓ |
| Examination | NIH Toolbox picture sequence age 7+ v2.1 |  | ✓ | ✓ |
| Laboratory study | Procalcitonin | ✓ |  | ✓ |
| Laboratory study | EBV anti early antigen IgG, viral capsid IgM, viral capsid IgG, nuclear antigen IgG |  | ✓ | ✓ |
| Laboratory study | Anti-nuclear antibody |  | ✓ | ✓ |
| Laboratory study | Anti-cyclic citrullinated peptide antibodies |  | ✓ | ✓ |
| Laboratory study | Rheumatoid factor |  | ✓ | ✓ |
| Laboratory study | Anti-dsDNA antibody | ✓ | ✓ | ✓ |
| Laboratory study | Ro antibody | ✓ | ✓ | ✓ |
| Laboratory study | La antibody | ✓ | ✓ | ✓ |
| Laboratory study | Smooth muscle antibody | ✓ | ✓ | ✓ |
| Laboratory study | RNP antibody | ✓ | ✓ | ✓ |
| Laboratory study | D-dimer | ✓ | ✓ | ✓ |
| Laboratory study | Anti-phospholipid antibody | ✓ |  |  |
| Laboratory study | Troponin |  | ✓ | ✓ |
| Laboratory study | N-terminal pro-brain natriuretic peptide |  | ✓ | ✓ |
| Laboratory study | Adrenocorticotropic hormone and morning cortisol |  | ✓ | ✓ |
| Laboratory study | Hepatitis B and C testing |  | ✓ | ✓ |
| Laboratory study | Parathyroid hormone | ✓ |  | ✓ |
| Laboratory study | Gamma-glutamyl transferase | ✓ |  | ✓ |
| Laboratory study | Cytokine panel (IL2 receptor; IL 1beta, 2, 4-6, 8, 10, 13, 17; interferon gamma, TNF alpha) | ✓ | ✓ | ✓ |
| Laboratory study | Supine and upright catecholamine testing | ✓ |  | ✓ |
| Laboratory study | ICAM-1 | ✓ | ✓ | ✓ |
| Laboratory study | Insulin | ✓ | ✓ | ✓ |
| Laboratory study | c-peptide |  |  |  |
| Laboratory study | Oral glucose tolerance test (time points 0, 30, 60, 120 min) | ✓ | ✓ | ✓ |
| Laboratory study | Fecal WBC | ✓ | ✓ | ✓ |
| Laboratory study | Fecal SARS-CoV-2 viral load (viral RNA and/or antigen) | ✓ |  | ✓ |
| Radiology | MRI brain with gadolinium | ✓ |  |  |
| Radiology | Volumetric non contrast chest CT (with inspiratory/expiratory scans) | ✓ | ✓ |  |
| Radiology | Dual energy chest CT with contrast |  | ✓ |  |
| Radiology | Resting transthoracic echocardiography with strain imaging | ✓ | ✓ | ✓ |
| Radiology | Renal ultrasound |  | ✓ | ✓ |
| Radiology | Fibroscan | ✓ | ✓ | ✓ |
| Radiology | Non-contrast abdominal CT | ✓ |  |  |
| Procedure | Electrocardiogram | ✓ | ✓ | ✓ |
| Procedure | Spirometry, resting SpO2 and single breath diffusion capacity | ✓ | ✓ | ✓ |
| Procedure | Tilt table test | ✓ |  | ✓ |

CT: computed tomography; MRI: magnetic resonance imaging

* The target window for performance of Tier 2 assessments is within 90 days of the date the study was triggered. Assessments not performed within that window are ineligible for completion; however, if the participant is still symptomatic and retriggers the assessment, or retriggers the assessment randomly, the study may then be performed within the new triggered window.
