# Supplementary material for: Researching COVID to Enhance Recovery (RECOVER) adult study protocol: Rationale, objectives, and design
Source: PLoS One. 2023 Jun 23;18(6):e0286297. doi: 10.1371/journal.pone.0286297 (PMC10289397; doi:10.1371/journal.pone.0286297)
Supplement: S5 Table — (DOCX) [file pone.0286297.s007.docx]

**S5 Table: Tier 3 Assessments**

| **Category** | **Assessment** | **In original protocol Tier 3** | **In current protocol Tier 3 (v7.0)** | **Performed in pregnant, <3 month post-partum** |
| --- | --- | --- | --- | --- |
| Examination | Michigan Neuropathy screen | ✓ |  | ✓ |
| Examination | Utah Early Neuropathy scale | ✓ |  | ✓ |
| Examination | Complete eye examination including optical coherence tomography | ✓ | ✓ | ✓ |
| Examination | ENT examination | ✓ |  | ✓ |
| Examination | Audiometry | ✓ | ✓ | ✓ |
| Examination | Complete neurocognitive testing (to include Digit span forward/backward; Trailmaking A (3 min); Trailmaking B (3 min); Hooper Visual Organization Test, MOCA/blind MOCA, multilingual naming test, phonemic test, Benson complex figure copy, CERAD word list, Craft Story 21 Recall)+ | ✓ | ✓ | ✓ |
| Examination | Endopat testing | ✓ | ✓ | ✓ |
| Laboratory study | Serum protein immunofixation electrophoresis | ✓ | ✓ | ✓ |
| Laboratory study | Serum B12 with methylmalonic acid | ✓ | ✓ | ✓ |
| Laboratory study | CPK, aldolase, myositis panel | ✓ | ✓ | ✓ |
| Laboratory study | Neurofilament light chain | ✓ | ✓ | ✓ |
| Laboratory study | Fecal SARS-CoV-2 viral load (viral RNA and/or antigen) | ✓ |  | ✓ |
| Laboratory study | Fecal calprotectin | ✓ | ✓ | ✓ |
| Laboratory study | Total Tau (single molecule array SIMOA) | ✓ | ✓ | ✓ |
| Laboratory study | Anti Mullerian hormone |  | ✓ | ✓ |
| Radiology | MRV/MRA brain | ✓ |  | ✓ |
| Radiology | MRI brain with gadolinium |  | ✓ |  |
| Radiology | MRI cervical spine with and without gadolinium | ✓ |  |  |
| Radiology | MRI thoracic spine with and without gadolinium | ✓ |  |  |
| Radiology | MRI lumbar spine with and without gadolinium | ✓ |  |  |
| Radiology | Dual-energy chest CT with contrast | ✓ |  |  |
| Radiology | Chest CT pulmonary angiography | ✓ |  |  |
| Radiology | Ventilation/perfusion scan | ✓ |  |  |
| Radiology | Cardiac imaging with meta-iodobenzylguanidine (mIBG)* | ✓ | ✓ |  |
| Radiology | Cardiac MRI, with and without gadolinium contrast | ✓ | ✓ |  |
| Radiology | Abdominal CT with contrast | ✓ |  |  |
| Radiology | Gastric emptying study | ✓ | ✓ |  |
| Procedure | Tilt table testing with supine/upright catecholamine |  | ✓ |  |
| Procedure | Cardiovagal innervation testing |  | ✓ | ✓ |
| Procedure | Nerve conduction study | ✓ | ✓ |  |
| Procedure | Electromyography | ✓ | ✓ |  |
| Procedure | Skin biopsy | ✓ | ✓ |  |
| Procedure | Muscle biopsy | ✓ | ✓ |  |
| Procedure | Lumbar puncture | ✓ | ✓ |  |
| Procedure | Facility-based sleep study | ✓ | ✓ | ✓ |
| Procedure | Full cardiopulmonary exercise testing | ✓ | ✓ |  |
| Procedure | Plethysmography lung volumes | ✓ |  | ✓ |
| Procedure | Bronchoscopy | ✓ | ✓ |  |
| Procedure | Right heart catheterization | ✓ | ✓ |  |
| Procedure | Upper endoscopy | ✓ | ✓ |  |
| Procedure | Colonoscopy with or without biopsy | ✓ | ✓ |  |

* The target window for performance of Tier 2 and 3 assessments is within 90 days of the date the study was triggered. Assessments not performed within that window are ineligible for completion; however, if the participant is still symptomatic and retriggers the assessment, or retriggers the assessment randomly, the study may then be performed within the new triggered window.
