# Supplementary material for: Researching COVID to Enhance Recovery (RECOVER) adult study protocol: Rationale, objectives, and design
Source: PLoS One. 2023 Jun 23;18(6):e0286297. doi: 10.1371/journal.pone.0286297 (PMC10289397; doi:10.1371/journal.pone.0286297)
Supplement: S6 Table — (DOCX) [file pone.0286297.s008.docx]

## **S6 Table: Writing Committee**

| Leora I. Horwitz |
| --- |
| Tanayott Thaweethai |
| Shari B. Brosnahan |
| Mine S. Cicek |
| Megan L. Fitzgerald |
| Jason D. Goldman |
| Rachel Hess |
| Sally L. Hodder |
| Vanessa L. Jacoby |
| Michael R. Jordan |
| Jerry A. Krishnan |
| Adeyinka O. Laiyemo |
| Torri D. Metz |
| Lauren Nichols |
| Rachel E. Patzer |
| Anisha Sekar |
| Nora G. Singer |
| Lauren E. Stiles |
| Barbara S. Taylor |

# 
