# Supplementary material for: Researching COVID to Enhance Recovery (RECOVER) adult study protocol: Rationale, objectives, and design
Source: PLoS One. 2023 Jun 23;18(6):e0286297. doi: 10.1371/journal.pone.0286297 (PMC10289397; doi:10.1371/journal.pone.0286297)
Supplement: S7 Table — (DOCX) [file pone.0286297.s009.docx]

# **S7 Table: RECOVER-Adult Consortium Members**

# **Atlanta RECOVER Clinical Research Site**

**Emory University**

*Ighovwera Ofotokun, PI*

*Rachel E Patzer, PI*

Rachael Abraham

Franchesca Aguilar

Quasheba (Yasmine) Allen

Blake Anderson

Casey Beaty

Chetna Bedi

Jasmine Berry

James Douglas Bremner

Shelby Collins

Angel Craft

D'Andrea Doyle

Jess Harding

Shilpa Krishnan

Hana Lee

Jose Daniel Leon

Dong Li

Christopher Martin

Atuarra McCaslin

Miranda Montoya

Caitlin Anne Moran

Tran Nguyen

Sofia Oviedo

ReNata Shaw

Cory Sylber

Larissa J Teunis

Kehmia Titanji

**Emory Healthcare (Hope Clinic)**

*Zanthia Wiley, PI*

Arijan Ager

Mary Atha

Mary Bower

Cartia Dixon

Rebecca Fineman

Nicole Martin Franks

Natalie Gray

Ash Grimes

Evan Gutter

Lisa Harewood

Lauren Nicole Hewitt

Christopher Huerta

Brandi Johnson

Lana Khalil

Dean Kleinhenz

Athena Koumanelis

Alexandra Koumanelis

Matthew Lee

Kennedy Lewis

Matthew Litvack

Nour Makkaoui

Eileen Osinski

Bernadine Panganiban

Dilshad Rafi Ahmed

Kazi Rahman

Paulina Alejandra Rebolledo

Nadine Rouphael

Talib Sirajud-Deen

Veronica Smith

Andre Stringer

Jessica Traenkner

Kristen Unterberger

Kris Varney

Dongli Wang

Erika Wimberly

Terra Jean Winter

**Grady Health System**

*Tiffany Walker, PI*

Walter Asencios

Donchel Boone

Ke'Ara Brown-Smith

Jannah Elchommali

Jenny Elizabeth Han

Cynthia Ifejika

Vidhi Javia

Jerrod McIntyre

Christopher Toy

Alex Truong

Tamara Wesley

**Morehouse School of Medicine**

*Priscilla Pemu, PI*

Carmel Alvarez

Kelechi Carl-Igwe

Annette Dandy

Carla Holloway

Monica Juarez

Jan Morgan-Billinslea

Elizabeth Ojemakinde

Michael Prude

Ruvina Silva

Cheryl Simpson

Ashley Sylvera

**Atlanta VA Health Care System**

*Sushma Komakula Cribbs, PI*

Ghazal Ahmadi-Izad

Alicarmen Alvarez

Gustavo Capo

Erna Clyburn

Julie Costello

Xiangqin Cui

Rijalda Deovic

Jeanne Dow

Anyssa Francis

Julia Gallini

Liliana Hernandez

Ketteria Ingram

Jordi Lainez

Vincent Charles Marconi

Loice Mbogo

Abeer Moanna

Elena Morales

Yolanda Paredes-Gaitan

Chantrice Rogers

Tehquin Tanner

Kodasha M Thomas

Kartavya Vyas

Juton Winston

Cherry Wongtrakool

**Kaiser Permanente of Georgia**

*Jennifer C. Gander, PI*

Patricia Bush

Jamila Johnson

Imanii Kolailat

Monica Martinez

Roslin Nelson

Robert B Neuman

Bukkie Ojoawo

Marni Segall

**Boston COVID-19 Recovery Cohort**

**Brigham and Women's Hospital**

*Bruce Levy, PI*

Nya Alexander

Lindsey Baden

Shamik Bhattacharrya

Julie Buring

Li Qing Chen

Cheryl Clark

Charles Czeisler

Lauren Donahue

Elizabeth Gay

Sheila Hegde

Adetoun Okenla

Bisola Ojikutu

Daniela Lamas

Kwabena Lartey

Nomi Levy-Carrick

Maureen Macgowan

Karen Magsipoc

JoAnn Manson

Susan Redline

Elizabeth Sampson

Haley Schram

Howard Sesso

Scott Solomon

Jeffrey Sparks

Maria Sundquist

David Systrom

Vivian Thu

David Walt

George Washko

Rebecca Weiner

Maureen Whittelsey

**Massachusetts General Hospital**

*Ingrid V. Bassett, PI*

George Alba

Galit Alter

Jasneet Aneja

Francesca Caramazza

Geoffrey Chen

Lilly Fernandes

Sarah Flannery

Mabelin Garcia De La Rosa

Joseph Giacino

Leo Ginns

Marcia Goldberg

Colin Goodbred

Jennifer Haas

Stephanie Horsfall

Surabhi Iyer

Elena Jin

Michelle Jones

Boris Juelg

Diane Kanjilal

Arthur Kim

Jodi Kurtz

Gregory Lewis

Valeria Magallan

Kristin Meader

Winna Mowenn

Taing Nandi Aung

Daniel Pacella

Roy Perlis

Jonathan Rosand

Ashley Stuckwisch

Anisha Tyagi

Zachary Wallace

Dean Xerras

Danielle Zionts

**Beth Israel Deaconess Medical Center**

*Janet Mullington, PI*

Toluwanimi Ajayi

David Alsop

Esther Apraku

Michelle Beck

Diara Canton

Ai-Ris Collier

Andrea Collins

Rammy Dang

Sourbha Dani

Ramona Faris

Tamara Fong

Wilanda Gabriel

Sarju Ganatra

Monika Haack

Halle Hall

Kristine Hauser

Wendy Hori

Matcheri Keshavan

Elizabeth LaSalvia

Andrew Lewis

Chun Lim

Jason Maley

Edward Mercantonio

Murray Mittleman

Rita Monahan

Keishi Nambara

Emily Peachthong

Yuri Quintana

Uyen Rasphoumy

Marjorie Rowe

Jennifer Scott-Sutherland

Lynn Shaughnessy

Kathryn Stephenson

Jennifer Stevens

Gyongyi Szabo

Andrew Taylor

Siline Thai

Robert Thomas

Michael Vazquez

James Wareing

Huan Yang

Oscar Yang

**Boston University/Boston Medical Center**

*Jai Marathe, PI*

Tracy Battaglia

Anna Cervantes-Arslanian

Elizabeth Duffy

Naomi Hamburg

Misaki Kobayashi

George O’Connor

Fitzgerald Shepherd

Charles Williams

**Cambridge Health Alliance**

*Janice John, PI*

Pieter Cohen

Geetika Gupta

Margaret Lanca

Amberly Ticotsky

**Tufts Medical Center**

*Michael Jordan, PI*

*Honorine Ward, PI*

Rebecca Badore

Deborah Blazey-Martin

Renee Brody

Maher Ghamloush

Vidya Iyer

Laura Kogelman

Marvin Konstam

Bipin Malla

Olaniyi Ogundobede

Debra Poutsiaka

Rupali Ranade

Dulia Santos

Paul Summergrad

David Thaler

Lauren Tobias

**South Shore Hospital**

*Frank Schembri, PI*

Paola Castellotti

Carolyn McLaughlin

Justin O'Leary

Simone Wildes

**Deep South SARS-CoV-2 Recovery Cohort**

**University of Alabama at Birmingham**

*Jeanne Marrazzo, PI*

*Nathan Erdmann, mPI*

*Emily Levitan, mPI*

Donna Armstrong

Kenneth Blackwell III

Annalia Causey

Felice Cook

Julio Domingo

Conner Donahue

Maitlyn Eady

Jeffrey Edberg

Susan Ellen Binkley

Melissa Garner

Brandon Gray

Wanda Hall

Cady Hart

Bertha Hidalgo

Kaylen Holtzapfel

Alexis Jinright

Suzanne Judd

Teri Kennedy

Leigh Kirkwood

Megan Maier

Patricia McCormack

Kevin Mitchell

Aoyjai Montgomery

Juan Pablo Pilco

Leigh Powell

Rachael Shevin

Sidney Skipworth

Leah Spurgeon

Gregory Ware

Rosanne Wilson

Dana Woodruff

**University of South Alabama**

*Mark Gillespie, PI*

Noah Garcia-McClaney

Jamie Hansel

Jing Wu

**University Medical Center New Orleans**

*Jyotsna Fuloria, PI*

Paula Datri

Michael Hagensee

Cathryn Leggio

Allen Perkins

Amber Trauth

Siobhan Trotter

Alexander Van Deerlin

Sharon Weiser

Madeline Young

**Louisiana State University**

*Lucio Miele, PI*

Todd Brown

Erica Sutherland

**Howard University**

**Howard University**

*Hassan Ashktorab, PI*

*Hassan Brim, PI*

*Adeyinka Laiyemo, PI*

*Zaki Sherif, PI*

Emmanuel Baidoo

Chioniso Jakazi

Alem Mehari

Julius Ngwa

Monique Perret Gentil

Ruth Quartey

Akbar Solemani

**University of Maryland Mercy Medical Center (UMD-MC)**

*Paul Thuluvath, PI*

*Anurag Maheshwari, PI*

Mhret Alemu

Jordan Anderson

Mahak Chauhan

Sung Cho

Karli Goodman

Gandi Lanke

Ralph Lebron

Jina Ok

Polly Robarts

Somya Shesadri

Chau To

Hwan Yoo

**IDeA States Consortium for Clinical Research (ISCORE)**

**West Virginia University**

*Sally Lynn Hodder, PI*

James Bardes

Connie Cerullo

Michelle Chidester

Renee Clark

Sherri Davis

Cynthia Duda

Chad Glaze

Cami Handlan

Paige Harman

Shelly Howenstein

Karen Hunter

Shruti Jaiswal

Joy Juskowich

Wesley Kimble

Stacey Koontz

Tanya Moran

Anne Oravets

Rebecca Reece

Meghan Reeves

Zhili (Julie) Shao

Shelley Welch

Haixia Tracy Yang

Suhil Zia

**Hispanic Alliance for Clinical and Translational Research**

*Carlos A. Luciano, PI*

Aracelis Arroyo

Ileana Boneta

Daniel Casiano

Sylvia Davila

Nilda Gonzalez

Marielly Lopez

Carola Lopez-Cepero Roman

Mariela Maisonet-Alejandro

Rene Marty

Mary Mays

Yaritza Moran

Litza N Pabon Malave

Sigrid Perez Frontera

Adelma Rivera

Ana C. Sala Morales

Waleska Sanchez-Vazquez

Jorge Santana

Ruth Santos

**LSU Health Sciences Center New Orleans**

*Judd Shellito, PI*

Holli Bologna

Virginia Garrison

Erin Meyaski

Mary Meyaski-Schluter

**LSU Pennington Biomedical Research Center**

*John P. Kirwan, PI*

Kimberly Banks

Emily Rachal

Emily Bebler

Grace Bella

Alexa Bennett

Erica Bertrand

Kara Devall

Gabriela Dominguez

Amber Dragg

Angela Eldredge

Elisabeth Fontenot

Greta Fry

Bethany Gildersteeve

Sara Goff

Frank Greenway

Lauren Harrington

Erin Herrin

Lisa Jones

Erin King

Leigh Lamonica

Stephen Lee

Yejee Lee

Robert Leonhard

Jennifer Levatino

Donald Lewis

Melissa Lingle

Angrielle Lloyd

Raoul Manalac

Brian Melancon

Carla Milo

Ronald Monce

Blair Pucheu

Jennifer Rood

Stacey Roussel

Connor Sanford

Monica Santos

Crissy Sharpe

Mandy Shipp

Brooklyne Smith

Aryelle Stafford

Amy Thomassie

Celeste Waguespack

Katherine Walgamotte

Aubrey Windham

**MaineHealth/Northern New England IDeA CTR**

*Clifford J. Rosen, PI*

Abigail Arruda

Emily Berg

Anne Breggia

Kathryn Brouillette

Ivette F. Emery

Marc Flore

Lindsey Gower

Teresa Martel

Lauren Moore

Darlene Peterson

Theresa Roelke

David Seder

**Sanford Health**

*Marc D. Basson, PI*

Nicole Arbach

Lora Black

Reagan Byrum

Susan Hoover

Jessica Just

Lindsay Krause

Debra Langstraat

Allison Lutz

Sara Onnen

Vanessa Williams

**Tulane School of Medicine**

*Vivian Fonseca, PI*

Emily Callegari

Shaveeta Gupta

Elvia Haynes

Roxanne Johnson

Neda Khoshkhoo

Brian Logarbo

Michele Longo

Sofia Marquez

Roberta McDuffie

Arianna Mohiuddin

Dynte Moore

Ahona Mukherjee

Aneisha Simon

Monica Smith

Neha Upadhyay

Blaire Williams

Mei Yang

**University of Hawaii/Mountain West CTR**

*Cecilia M. Shikuma, PI*

Dominic Cheung Chow

Boonyanudh Jiyarom

Bradley Mak

Eduardo Manzano

Grace Matsuura

Cris Milne

Lorna Nagamine

Debbie Ogata-Arakaki

Rachel Ouye

Victoria Rivera

Zao Zhang

**University of Kansas Medical Center**

*Mario Castro, PI*

Charles Bengtson

Chris Bessmer

Luigi Boccardi

Jonathan Boomer

Maggie Chen

Kenton Felmlee

Theresa Howard

Jaehun Jeong

Kathy Jurius

Pamela Kemp

Christina Pantalunan

Tyler Re

Adam Ruff

Betina Senat

Leah Seymour

Leslie A. Spikes

Jennifer Troyer

Vanessa Verschelden

Vianca Williams

**University of Kentucky**

*Sidney Waldo Whiteheart, PI*

Hammodah Alfar

Suzanne Arnold

Laura Ashe

Marietta Barton-Baxter

Ryleigh Board

Suzanne Burchett

John Peyton Bush

Beth Garvy

Olivia Hage

Ellen Hartman

Darren Henderson

Melissa Hollifield

Frazier Moore

James Zachary Porterfield

Martha Sim

Ryan Weeks

Jeremy Wood

**University of Mississippi Medical Center**

*Gailen Marshall, PI*

Robert Brodell

Jamie Brown

Donielle Drakes

Vishnu Garla

Sarah Glover

Michael Hall

Thomas Hudson

Kia Jones

Christopher Moore

Rachel Morris

Utsav Nandi

Kelsey Napper

Bhagyashri D. Navaleke

Leila Seidfaraji

Shashank Shekar

John Spurzem

Amy Wigglesworth

**University of Nebraska Medical Center**

*Andrew Vasey, PI*

Amira Abdus-Salaam

Karen Blessing

Daniel Copeland

Kristi DeHaai

John D Dickinson

Irena Kovacevic

LuAnn Larson

Tracy Mathisen

Marah Miller

Kristen Nickolas

Katie Ostlund

Mary Peguero

Vaishali Phatak

Alex Reed

Maria Thurow

David E. Warren

Sara Warta

Koreen Wede

Abigail Zatkalik

**University of Oklahoma Health Sciences Center**

*Judith A. James, PI*

*Timothy VanWagoner, PI*

Cristina Gale Arriens

Amanda L. Bogie

Cathy Carmichael

Brittany Karfonta

Geneva Marshall

Tiffany Moore

Valorie Owens

James Scott

Fatima Sukhera

Timothy Walsh

Drake Williams

**ILLInet**

**University of Illinois Hospital & Clinics**

*Jerry A Krishnan, PI*

Neha Atal

Aileen Baker

Sunni Barbera

Rachel Beety

Daksh Bhargava

Andrew D. Boyd

Taylor Breiter

Elizabeth Calhoun

Ruby Camacho

Michael Carrithers

Lauren Castro

Gabrielle Cavaliere

Rashmika Chalamalla

David Chestek

Tabitha Chettupally

Judith A. Cook

Dawood Darbar

Raktima Dasgupta

Marina Del Rios

Julie DeLisa

Sai Dheeraj Illendula

Kathleen Diviak

Tara Driscoll

Mark Steven Dworkin

Angela M. Ellison

Clarie Flanigan

Meghan Fortune

Meghan Fortune Donlon

Divya Francis

Michael Freedman

Lynn Gerald

Wayne H. Giles

Bayan Hammad

Sharon Hasek

Wendy Hasse

Maryann Holtcamp

Martyna Hryniewicka

Bryan Huerta

Nina Huynh

Niha Idrees

Nahed Ismail

Akash Jain

Kyle Jennette

Shrinidhi Kadkol

Grace Kadubek

Denise Kent

Jonathan D. Klein

Lucia Large

James Lash

Michele Ledbetter

James Lee

Cinthia Leman

Jun Lu

Miriam Martinez

Cammeo Mauntel-Medici

Amy McManus

Conny Mei

Martha Menchaca

Robin J. Mermelstein

Tennessee Miller

Abeer Mohamed

David Moreno

Liam Morrissy

Naoko Muramatsu

Hugh Musick

Lourdes Norwick

Richard M. Novak

Elizabeth Ochoa-Raya

John O'Keefe

Abigail Olsen

Khushboo Patel

Nicolas Perez

Neil Pliskin

Erin Pozzolano

Bellur S. Prabhakar

Bharati Prasad

Barbara Predki

Heather M. Prendergast

John G. Quigley

Ramaswamy Ramchandran

Sarah Rappe

Ann Roach

Matthew Rowley

Gowrisree Rudraraju

Melissa Rutherfoord

Jason Scheer

Tina Schuh

Jennifer Sculley

Nancy Shapiro

Jerisha Smith-Mack

Lauren Speakman

Nancy Tartt

Angela Tobin

Ellen Uppuluri

Melissa Uribe

Terry L. Vanden Hoek

Laura Villanueva

Ceolamar Ways

**BrightStar Community Outreach**

Kathy Cullick

Pastor Chris Harris

Carl Hearne

Joani Vaughan

Erron Williams

**Chicago Urban League**

Calmetta Coleman

Lela Olds

**Envision**

Adriana Mateo

Jennifer Ramirez

**Illinois Unidos**

Lisa Aponte-Soto

Javier Arellano

Maya Diaz

Hilda Garcia

Saira Garcia

Alejandra Ibañez

Marilyn Ortiz

Cesar Rolon

Maite Zapata

**Mile Square Health Centers**

*Janet Y. Lin, PI*

Judes Fleurimont

**OSF Healthcare/St. Francis Medical Center**

*John Hafner, PI*

Isidra Baker

Jennifer Bandy

Dawn Bolliger

Praneeth Chebrolu

Jennifer Dixon

Michael Downey

Lisa Gale

Keith Hanson

Kimberly Hartwig

**Peoria City/County Health Department**

Monica Hendrickson

Seth Noland

Tracy Terlinde

**Teamwork Englewood**

Brianna Hobbs

**UI College of Medicine Peoria**

*Sarah Donohue, PI*

Sara Kelly

Jerusha Boyineni

Hannah Curry

Sherrie Edmonds

Phoebe Maholovich

Sergey Malchenko

Sarah Stewart de Ramirez

Tiffany Thompson

**UnityPoint Health**

*Samer Sader, PI*

Savannah Cranford

Terri Osmulski

Praveen Sudhindra

**Mount Sinai PASC Coalition (SinaiPACT)**

**Icahn School of Medicine at Mount Sinai**

*Alexander Charney, PI*

*Girish Nadkarni, PI*

*Juan Wisnivesky, PI*

Rawan Abdel Galil

Taj Adams

Steven Ascolillo

Esther Assenso

Emilia Bagiella

Jacqueline Becker

Kirk Campbell

Adrien Canery

Erica Cha

Esther Cheng

Dayeon Cho

Alyssa Civil

Carlos Cordon-Cardo

Chase Gornbein

Kaberi Dhar

Andrew Draheim

Jeremiah Faith

Zahi Adel Fayad

Brian Fennessy

Kaleigh Fidaleo

Sacha Gnjatic

Caroline Goldstein

Ruchir Goswami

Malika Gregory

Sabina Guliyeva

Lori Harvey-Ingram

Carol Horowitz

Rachel Jackson

Minal Kale

Matthew Hill

Jillani Kamran

Seunghee Kim-Schulze

Srisundesh Kodali

Jonathan Koganov

Amy Kontorovich

Patricia Kovatch

Jennifer Kucera

Jenny Lin

Inna Lishchenko

Martina Lopez May

Kathryn Marcon

Robert Marvin

Megan McVeety

Miriam Merad

Dara Meyer

Janice Morinigo

Khadeja Moses

Maya Nussenzweig

Cindy Osei

Tiffani Padua

Anisha Pal

Louis Pasquale

Vibhuti Patel

Farah Rahman

Michelle Ramos

Lynne Richardson

Leah Samuels

Jewelle Schofield

Joyce Serebrenik

Abdullah Serri

Nicole Simons

Sarah Tsuruo

Akosua Twumasi

Jaclyn Verity

Einab Weingarten

Alexis Whitaker

Lillian Wilkins

Michell Yee

Kimia Ziafat

**Mountain States PASC Collaborative**

**University of Utah**

*Rachel Hess, PI*

Haddy Bah

Macy Barrios

Conner Beckstead

Grayson Charlton

Dagny Donohue

Amanda Edwards

Julio Facelli

Isaac Ford

Dylan Freston

Reese Gorey

Tina Greimes

Paul Hartman

Ainsley Huffman

Alfonso Lopez

Juliemar Medina

Sophia Perez

Tristan Planelles

Jenny Powell

Annie Risenmay

Laura Scarborough

MaryBeth Scholand

Suzanne Vernon

**Denver Health and Hospital Authority**

*Edward M. Gardner, PI*

Tanner Bryan

Kaitlin Buck

Kellie Hawkins

Amy Irwin

Judy Oakes

Cole Ossian

**Intermountain Healthcare**

*Kirk Knowlton, PI*

Bailee Aguirre

Jeff Anderson

Kayelee Auer

Tami Bair

Lindsay Bosh

Lorlie Evans

Chase Garrett

Dixie Harris

Katherine Herrera

Eliotte Hirshberg

Ben Horne

James Juan

Stacey Knight

Lindsay Leither

Heather Maestas

Heidi May

Gabriel Najarian

Darija Runjaic-Ward

Scott Woller

Shyanne Zubal

**University of Colorado Anschutz Medical Campus**

*Kristine Mace Erlandson, PI*

Natasha Altman

Jill Bastman

Nick Charron

Jamie Cronin

Jesse Cwik

Elen Feuerriegel

Lani Finck

Adit Ginde

Paige Graham

Amy Harjes

David Huynh

Brandon Johnson

Sarah Elizabeth Jolley

Marjorie McIntyre

Jeffrey McKeehan

Aaron Mobley

Sarah Montoya

John Nguyen

Priscilla Pando

Kayleigh Reid

Ron J. Sokol

Shelby West

**University of New Mexico Health Sciences Center**

*Hengameh Raissy, PI*

David Archuleta

Rebecca Brito

Elyce Sheehan

Noella Garcia-Soberanez

Frederick Gentry

Eve Gronert

Michelle Sue Harkins

Debbie Lovato

Noah Martinez

Lorenzo Montoya

Alisha Parada

Davin K. Quinn

Alfredo Ramos

Irena Treacher

**NorthEast Ohio Covid United for REcovery (NEO-CURE)**

**Case Western Reserve University/University Hospital**

*Grace McComsey, PI*

Sadeer Al-Kindi

Rita Abbud

John Andrefsky

Alexis Baloi

Nicholas Boldt

Melinda Caraballo

Mary Chong

Kathryn Clark

Ann Conrad

Mary Consolo

Rebecca Curtis

Lynette Curtis

Margaret Clapp

Brian D’anza

Sarah Dawson

Kathryn DiFrancesco

Jared Durieux

Ebenezer Eteshola

Jihane Faress

Michelle Gallagher

Olivia Giddings

Paul Harris

Carla Harwell

Carla Hernandez

Shirin Iqbal

Olivia Kennedy

Danielle Labbato

Antonio Levert

Jennifer Levin

Angelica Levreault

Christian Mouchati

Caleb Mavar

Keely Newson

Princess Ogbogu

Morgan Pulling

Laura Puskas

Amanda Rivera

Michael Rodgers

Theresa Rodgers

David Rosenberg

Arnab Roy

Sarah Scott

Niyati Sheth

Beth Smith

Megan Tribout

George Yendewa

David Zhang

Sokratis Zisis

**The MetroHealth System**

*Nora Singer, PI*

Mohammed Abuzahrieh

Mirna Ayache

Emma Barnboym

Hailey Chesnick

Marissa Edminston

Larraine Gordesky

Carla Greenwood

Maricela Haghiac

Elizabeth Kaufman

Rebecca Lowenthal

Ketrin Lengu

Bridget Mackin

Shahdi Malakooti

Judy Minium

Christine Oleson

Ann Pearman

Kris Russ

Cheryl Smith

Terry Stancin

Daniel Temple

Elisheva Weinberger

**The Pacific Northwest Consortium for Post-Acute Sequelae of SARS-CoV-2 Infection**

**Institute for Systems Biology**

*James Richard Heath, PI*

Conor Brennan

Rick Edmark

Simon Evans

Vanessa Gutierrez

Jennifer Hadlock

On Ho

Kathleen Jade

Sarah Li

Andrew T. Magis

Michaela McKasson

Lee Rowen

Thea Swanson

Dan Yuan

**Cedars-Sinai Medical Center - Los Angeles, CA**

*Peter Chen, PI*

Antonina Caudill

Susan Jackman

Brittany Mattison

Sam Torbati

**Providence Regional Medical Center Everett**

*George Diaz, PI*

Angela Berrios

Jerome Differding

Vanessa Elan

Keely Heredia

Nasiha Hussain

Jo Joslin

Courtney Rinehart

Rebecca Watson

**Providence Sacred Heart Medical Center**

*Katherine Tuttle, PI*

Radica Alicic

Joni Baxter

Lisa Davis

Sarah Emerson

Claudia Flores

Susan Hood

Kelli Kuykendall

Allison Lambert

**Swedish Health Services/ISB/Swedish Medical Center**

*Jason D. Goldman, PI*

Heather Algren

Aaron Ayenew

James Del Alcazar

Allie Duven

Stephanie Johnson

John Kaneko

Christina Kim

Paula Manner

Marisa McCormack

Tija Tippett

Julie Wallick

Natalie Young

**University of Washington**

*Helen Y. Chu, PI*

Eric Chow

Nicholas Franko

Emily Guthrie

Megan Kemp

Jennifer Logue

Denise McCulloch

Dylan McDonald

Callista Nackviseth

**PREVAIL South Texas**

**University of Texas Health Science Center at San Antonio**

*Thomas F. Patterson, PI*

*Barbara S. Taylor, PI*

Reed Anderson

Azaneth Arellanes

Rose Ann Barajas

Cheryl Farner

Melinda Fischer

Mark P. Goldberg

Monica Verduzco-Gutierrez

Gabrielyd Hastings

Patricia Heard

Italia Herrera

Edgar Infante

Lisa Longoria

Hillary Johnson

Johnnie Jones

Emeka Okafor

Jan Evans Patterson

Alexis Pinones

Jennifer Potter

Brian Reeves

Irma Scholler

Sudha Seshadri

Dimpy Shah

Pankil Shah

Bridgette Soileau

Pamela Solis

Carmen Stoebner

Michael Sullivan

Robin Tragus

Joel Tsevat

**UT Education and Research Center at Laredo**

*Claudia Castillo Paredes, PI*

Stephanie Alvarado

**Stanford Post-Acute Recovery Cohort (SPARC)**

**Stanford University**

*Upinder Singh, PI*

*Paul Utz, PI*

Aubrey Adio

Neera Ahuja

Shuchi Anand

Leonard Basobas

Catherine Blish

Andra Blomkalns

Jenna Bollyky

Hector Bonilla

Athanasia Boumis

Richard Brotherton

Kimberly Clinton

Liisa Dewhurst

Vaidehi Dingankar

Julia Donahue

Jorge Doranets

Jinelle Fields

Linda Geng

Rojin Ghobadi

JaVahn Iverson

Karen Jacobson

Prasanna Jagannathan

Yasmin Jazayeri

Kathryn Jee

Jaliza Johnson

Celeste Jupiter

Ryan Kelley

Naresh Khurana

Charity Kim

Andre Kumar

Amy Kuo

Jamison Langguth

Kharma Lhamo

Martina Madrigal

Chaitasi Majmudar

Yvonne Maldonado

Lisa Maredia

Ellen O'Conor

Nicole Odenwald

Andrew T. O'Donnell

Divya Pathak

Rinoka Sato

Allyson Tayag

Crystal Ton-Nu

Mary Rithu Varkey

Anita Visweswaran

Samuel Yang

**Stanford Tri Valley**

*Minjoung Go, PI*

Christopher Jamero

Xiaolin "Kathleen" Jia

Kelly Olszewski

Orlando Quintero

Jake Scott

**United Against COVID - AZPC3 Consortium**

**University of Arizona**

*Janko Nikolich-Zugich, PI*

*Sairam Parthasarathy, PI*

Carly Deal

Rodriguez Esquival Denise

Isabella DuPre

Kacey C. Ernst

Lunar Far

Lucia Felix

Mariana Felix

Angelica Galdamez-Avila

Amanda E Galster

Omar Gomez

Edgar Gutierrez

David T. Harris

Stefanie Harris

Adrianna Hernandez

Michael Hernandez

Maria Karnafel

Colleen Kenost

Maria Khawam

Alison Koleski

Suhr Kyle

Bonnie LaFleur

Brenda Lambert

Sicily LaRue

Ryan Lee

Kylie Lew

Karen Lutrick

Nirav Merchant

Christopher Morton

Sabrina OesterleHas

Toluwanimi Olorunnisola

Courtney L Olson

Jeanette Peralta

Iliana Perez

William (Vern) Pilling

Kristen Pogreba-Brown

Eric Reiman

Denise Rodriguez Esquivel

Megan Rumble

T. Lee Ryan

Erick Sanchez

Maria Santa Cruz

Terry Smith

Manuel Snyder

Vignesh Subbian

Kyle Suhr

Nancy K. Sweitzer

Annie Van Den Broeke

Deanna Velarde

April Yingst

**Banner University Medical Center (BUMC) Phoenix**

*Joyce Lee-Iannotti, PI*

Lynn Autry

Sabine Borwege

Maura Carriel

Jacquelynn Copeland

Marjorie DiLise-Russo

Susan Fadden

Marilyn Glassberg

Isaias Gomez

Garrett Grischo

William Hartley

Leah Hillier

Harvey Hsu

Hira Ismail

Stephanie Iusim

Michelle James

Mrinalini Kala

Erika Kenney

Daniel Kim

Kenneth S. Knox

Melanie MacNevin

Nicole Marshell

James Michelle

Ganesh Murthy

Jami Ochoa

Fatima Palacios

Elizabeth Quijada

Elizabeth Rawnsley

Elizabeth Russo

Christina Sosa

Samuel Unzek

Sheila Vadovicky

Sharry Veres

**Banner University Medical Center (BUMC) Tucson**

*Eric M. Reiman, PI*

Maria Ambrose

Christian Bime

Joy Elizabeth Bulger Beck

Tiffanie Cagle

Austin Derma

Margaret Drury

Damien Duran

Jose Elizondo

Rebecca Foley

Brent Gary

Claudia Gonzales

Lillian Hansen

Trina Hughes

Ayesha Javed

Elizabeth Juneman

David Lieberman

Margaret McCann

Jarrod Michael Mosier

Katie Raymer

Nikki Reed

Franz Rischard

Tanya Sandhu

Francisco Soto

Carlos Tafich-Rios

Uma Reddy

Cathleen Wilson

**Mayo Clinic Scottsdale**

Chyke Abadama Doubeni

**University of California San Francisco**

**University of California San Francisco**

*Steven Deeks, PI*

*Dan Kelly, PI*

*Jeffrey Martin, PI*

*Michael Peluso, PI*

Khamal Anglin

Urania Argueta

Kofi Asare

Amethyst Belanger

Melissa Buitrago

Aimee Cantoran

Celina Chang Song

Alexus Clark

Nicole Del Castillo

Monika Deswal

Matthew Durstenfeld

Halle Grebe

Timothy Henrich

Rebecca Hoh

Priscilla Hsue

Beatrice Huang

Billy Huang

Rania Ibrahim

Pamela Josue

Marian Kerbleski

Raushun Kirtikar

Salman Mahboob

Sadie Munter

James Lombardo

Monica Lopez

Michael Luna

Carina Marquez

Lynn Ngo

Randy Parada

Kimberly Rhoads

Antonio Rodriguez

Alma Rodriguez Lopez

Justin Romero

Dylan Ryder

Matthew So

Viva Tai

Brandon Tran

Daisy Valdivieso

Deepshika Verma

Meghann Williams

Andhy Zamora

## **Adult Pregnancy Cohort**

**PRIORITY: Post-Acute Sequelae of SARS-CoV-2 in Pregnant Women and their Children**

**University of California San Francisco**

*Vanessa Jacoby, PI*

Nyat Araya

Cinthya Arellano-Melchor

Ann Chang

Isabel De La Torre

Soujanya Gade

Estefania Guerreros

Victoria Laleau

Vanessa Monzon

Marie Salem

Maria Tolentino

**MFMU PASC-PREG**

**University of Utah**

*Torri Metz, PI*

Brynlee Buhler

Noah Carson

Jacob Draper

Kevin Duff

Marie Gibson

Denise Lamb

Amanda Nelsen

Shannon Schlater

Amber Sowles

Cassandra Vance

**Brown University (Women and Infants Hospital)**

Marshall Baez

Lisa Beati

Donna Catlow

Angelica DeMartino

Shafaq Jawed

Diana Kuhn

Haley Lefebvre

Paula Lorenzi

Jane Milano

Stephanie Nunez

Amanda O’Neill

Athena Poppas

Dwight Rouse

Janet Rousseau

**Case Western - MetroHealth Medical**

*Jennifer Bailit, PI*

*Kelly Gibson, PI*

Wendy Dalton

Brittany DeSantis

Bailey Diaz

Parmjit Gill-Jones

Melissa Kinas

Joan Lippus

Brian Mercer

Judi Minium

Abigail Pierse

LuAnn Polito

Ava Reese

Eugenia Sweet

**ChristianaCare**

*Matthew Hoffman, PI*

Caitlin Almeida

Carrie Kitto

Shannon Traczykiewicz

Ashley Vanneman

**Columbia University**

*Uma Reddy, PI*

Sabine Bousleiman

Sheica Cedano

Sara Echeverri

Megan Loffredo

Rupa Ravi

Noelia Zork

**Duke University Medical Center**

*Brenna Hughes, PI*

Nixaliz Cumba

Jennifer Ferrara

Lena Fried

Danielle Lanpher

**The George Washington University**

*Rebecca Clifton, PI*

Katia Barrett

Greg Sandoval

Steven Weiner

**Good Samaritan**

*Mounira Habli, PI*

Marta McClellan

Beth Sears

**Medical College of Wisconsin**

*Anna Palatnik, PI*

Mariana Karasti

Johanna Kessel

Christina Meyer

Eleanor Saffian

**Miami Valley Hospital**

*Samantha Wiegand, PI*

Kathleen Fennig

David McKenna

Emily Reynolds

Esther Kaye Snow

Rebecca Wirth

**New York-Presbyterian/Queens**

*Daniel Skupsk, PI*

Giorgi Kvashilava

Rosalyn Chan-Akeley

Sara Lucia Echeverri

Andrea Perez

Kelly Zhou

**NorthShore University HealthSystem**

*Beth Plunkett, PI*

Kevin Hascher

Dina Kapogiannis-Politis

Kathy Kearns

David Ouyang

Sunitha Suresh

Areebah Waseem

**Northwestern University**

*Lynn Yee, PI*

Dequana Jones

Gail Mallett

Audrey McMahon

Emily Miller

Mercedes Ramos

Trista Reynolds

Isabel Uribe

**Ohio State University**

*Maged Costantine, PI*

Anna Bartholomew

Stephanie Brindle

Barbara Cackovic

Sommer Chaney

Dawn Cline

Alyson Johnson

Baylee Klopfenstein

Huban Kutay

Devra Mast

Kayla McDaniel

Alexis Neri

Melanie Paglione

Sounali Perez

Sydney Rentsch

Jessica Russo

Taryn Summerfield

Yan Yuan

Mark Landon

Stephen Thung

Cynthia Shellhaas

Michael Cackovic

Heather Frey

Kara Rood

Patrick Schneider

Kartik Venkatesh

Courtney Abshier-Ware

Erin Cleary

Jennifer Grasch

Miranda Kiefer

Mahmoud Abdelwahab

Joe Eid

Monique McKiever

Sophia Andreatta

Christine Field

Caroline Bank

Xiao-yu Wang

Olivia Starcher

**Saint Peter's University Hospital**

*Kristy Palomares, PI*

Imene Beche

Danielle Graziano-Carrete

Clara Perez

Molly Sklios

**University of Alabama at Birmingham**

*Alan Tita, PI*

Nitin Arora

Kenneth Max Blackwell

Mariela Blair

Nicole Burrell

Lisa Dimperio

Donna Dunn

Janatha Grant

Madison Mann

Myriam Peralta

Lakia Pettibone

Jhana Plump

Jawan Struggs-Jemison

**University of Colorado**

*Camille Hoffman, PI*

Jocelyn Phipers

**University Hospitals MacDonald's Women's Hospital**

*David Hackney, PI*

Christopher Nau

**University of North Carolina - Chapel Hill**

*John Thorp, PI*

Kelly Clark

Inez Dufresne

Chelsea Grinnan

Molly Leatherland

Kathy Lloyd

Hannah Nunn

Sally Timlin

**University of Pennsylvania**

*Samuel Parry, PI*

Christina Fazio-Pizzi

Anna Filipczak

Emily Long

Meaghan McCabe

Abigail Roche

Haresh Sehdev

**University of Pittsburgh**

Jeanette Boyce

Reagan Devine

Francesca Facco

Sarah Hankle

Rachel Hines

Maura Hohn

Sharon Price

Kayli Rodgers

Marina Rushchak

Frank Sciurba

Hyagriv Simhan

Jason Styer

John Vargo

Sarah Whelan

**University of Texas HSC at Houston**

*Hector Mendez-Figueroa, PI*

Karen Castelan-Balbuena

Cynthia Edmonds

Luz Garcia

Adrienne Gross

Felecia Ortiz

Juanita Rugerio

Zina Spears

Jenifer Treadway

**University of Texas Medical Branch at Galveston**

*George Saade, PI*

Jennifer Cornwell

Luis Pacheco

Ashley Salazar

Lisa Thibodeaux

Jennifer DeVolder

**WakeMed**

*Carmen Beamon, PI*

**Yale University**

*Christian Pettker, PI*

Donna Allard

Sherrie Bitterman

Monika Lau

Jessica Leventhal

Lauren Perley

Linda Rink

## **Administrative Coordinating Center at Research Triangle Institute International**

*Lisa Newman, PI*

Quinn Barnette

Patricia Ceger

Mike Enger

Katie Fain

Tonya Farris

Sean Hanlon

David Hines

Kevin Jordan

Beth Linas

Meisha Mandal

Susan Nance

Lisa Newman

Claire Quiner

Rita Sembajwe

Gwendolyn Shaw

Vanessa Thornburg

Kendall Tosco

## **Clinical Science Core at NYU Langone Health**

*Rachel Gross, PI*

*Leora Horwitz, PI*

*Stuart Katz, PI*

*Andrea Troxel, PI*

Precious Akinbo

Ramona Almenana

Malate Aschalew

Lara Balick

Jasmine Briscoe

Shari Brosnahan

Alicia Chung

Stanley Cobos

Nakia Croft

Angelique Cruz Irving

Jasmin Divers

Shari Esquenazi-Karonika

Elias Febres

Catherine Freeland

Richard Gallagher

Jennifer Hossain

Neha Kansal

Tammy Kershner

Judy Kwak

Michelle F. Lamendola-Essel

Sarah Laury

Lei Lei

Janelle Linton

Max Logan

Nadia Malik

Gabrielle Maranga

Lia Mamistvalova

Maika Mitchell

Praveen C. Mudumbi

Erica Nahin

J.R. Rizzo

Johana Rosas

Chelsea Rose

Christina Saint Jean

Naomi Simon

Miranda Stinson

Mary Thomas

Lorna Thorpe

MeeLee Tom

Mmekom Udosen

Carlos Valencia

Jessica Velazquez-Perez

Crystal Vidal

Amy Willerford

Marion J. Wood

Shonna Yin

Susanna Zavlunova

## **Data Repository Core at Massachusetts General Hospital**

*Andrea Foulkes, PI*

*Elizabeth Karlson, PI*

*Shawn Murphy, PI*

Shifa Ahmed

Layne Ainsworth

Marie-Abèle Bind

Caryn Boehm

Mark Bohen

Natalie Boutin

Victor Castro

James Chan

Vivian Gainer

Randy Gollub

James Kerr

Doug MacFadden

Richard Morse

Amber Nguyen

Bridget Perry

Lynn Simpson

Ravi Thadhani

Tanayott Thaweethai

Nich Wattansain

Griffin Weber

## **PASC Biorepository Core at Mayo Clinic**

*Mine Cicek, PI*

Nancy Chang

Evan Ellingworth

Jordan Weyer

Jennifer Wheeler

Samantha Wirkus

Nicole Zahnle
