# Supplementary material for: Researching COVID to Enhance Recovery (RECOVER) adult study protocol: Rationale, objectives, and design
Source: PLoS One. 2023 Jun 23;18(6):e0286297. doi: 10.1371/journal.pone.0286297 (PMC10289397; doi:10.1371/journal.pone.0286297)
Supplement: S8 Table — (DOCX) [file pone.0286297.s010.docx]

## **S8 Table: RECOVER-Adult Committees and Task Forces**

## **Executive**

Hugh Auchincloss

Diana Bianchi

Joe Breen

Patti Brennan

Jeffrey Burns

Nakela Cook

Emily Cunningham

Felicia Davis Blakley

Betty Diamond

Mitchell S.V. Elkind

Tonya Farris

Lee Fleisher

Andrea Foulkes

Gary Gibbons

Laurie Gutmann

Michael Iademarco

Stuart Katz

Walter Koroshetz

Eldrin F. Lewis

Peter Marks

Hilary Marston

Mitchell Miglis

Lisa Newman

Tracy Nolen

Carlos A. Pardo-Villamizar

Amy Patterson

Sam Posner

Wendy S. Post

Serena Spudich

Clinton Wright

Heather Yates

Kanecia Zimmerman

**Steering**

Malate Aschalew

Audie Atienza

Charles Bailey

R. Graham Barr

Andra Blomkalns

Melissa Bondy

Hassan Brim

Jeffrey Burns

Alexander Charney

Benjamin Chen

Mine Cicek

John Crary

Dawood Darbar

Sean Deoni

Kathi Diviak

Ray Ebert

Jamie Elifritz

Amy Elliott

Robert L. Ferrer

John Fessel

Aloke Finn

Thomas Flotte

Andrea Foulkes

Emily Gallagher

Maria Gennaro

Rachel Gross

Melissa Haendel

James Heath

Rachel Hess

Sally Hodder

Carol Horowitz

Leora Horwitz

Vanessa Jacoby

Sarah Jolley

Suzanne Judd

Elizabeth Karlson

Stuart Katz

Rainu Kaushal

Lawrence Kleinman

Jerry Krishnan

Craig Lefebvre

Lei Lei

Emily Levitan

Bruce Levy

Daniel Liu

Jeffrey Martin

Grace McComsey

Julie McMurry

Robin J. Mermelstein

Torri Metz

Lucio Miele

Sindhu Mohandas

Janet Mullington

Shawn Murphy

Jane Newburger

Lisa Newman

Igho Ofotokun

Princess Ogbogu

Michelle Olive

Sairam Parthasarathy

Thomas Patterson

Priscilla Pemu

James (Zach) Porterfield

Antonello Punturieri

R. Ross Reichard

Jane Reusch

Kyung Rhee

Kathleen Rodgers

Juan Salazar

Lisa Schwartz-Longacre

Sudha Seshadri

Howard Sesso

Eyal Shemesh

Allan Shipp

Upinder Singh

Jessica Snowden

Serena Spudich

Cheryl Stein

Melissa Stockwell

James Stone

Jun Sun

Mehul Suthar

David Systrom

Brittany Taylor

Stephen Thibodeau

Andrea Troxel

PJ.Utz

Tiffany Walker

David Warburton

Gail Weinmann

Neely Williams

Dana Wolff-Hughes

John Wood

**Adjudication**

Khamal Anglin

Emilia Bagiella

Ryan Branski

Rodica Busui

Marissa Diggs

Vivian Gainer

Sunanda Gaur

Linda Geng

Sarah Jolley

Sarah Laury

Jai Marathe

Lisa McCorkell

Jarrod Mosier

Binita Shah

Dimpy Shah

Tiffany Walker

Peter Whitesell

**Ancillary Studies**

Hassan Ashktorab

Christine Bevc

Karyn Bishof

Yu Chen

Lori Chibnik

Dani Dumitriu

Jennifer Frontera

Paul Goepfert

Sylvie Goldman

Stephen Hewitt

Matt Huentelman

Barbara Karp

Jerry Krishnan

Marrah Lachowicz-Scroggins

Sarah Laury

Bruce Levy

Miriam Merad

Shawn Murphy

Janko Nikolich-Zugich

Laura Pace

Alice Perlowski

Brian Reeves

Juan Salazar

Sujata Thawani

Hannah Valantine

Drenna Waldrop

**Cardiopulmonary**

Natasha Altman

Soham DasGupta

Marissa Edminston

Josh Fessel

Aloke Finn

Tyler Gustafson

Francois Haddad

Jennifer Hossain

Priscilla Hsue

Pavitra Kotini-Shah

Sankaran Krishnan

Anu Lala-Trindade

Simon Lee

Alem Mehari

Patricio Millar Vernetti

Andre L Moreira

Anoop Nambiar

Robert Padera

Gail Pearson

Dhaval Raval

Franz Rischard

Erika Rosenzweig

Barbara Sampson

Frank Sciurba

Jackie Szmuszkovicz

Julie Thompson

Dongngan Truong

Viola Vaccarino

Alison Van Dyke

George Washko

John Wood

**Commonalities with Other Post-viral Syndromes**

Hector Bonilla

Christine Capone

Sekai Chideya-Chihota

Dane Cook

Walter Dehority

Monica Gutierrez

Rohan Hazra

Leonard Jason

Phillip Joseph

Dan Kelly

Joyce Lee-Ianotti

Vincent Marconi

Joshua Milner

Benjamin Natelson

Lisa O'Brien

Carlos Oliveira

James (Zach) Porterfield

Claire Quiner

Zaki Sherif

Nora Singer

Inderjit Singh

Jessica Snowden

David Systrom

C. Sabrina Tan

Emily Taylor

Vanessa Thornburg

Suzanne Vernon

**Core Operations Group**

Quinn Barnette

Frank Blancero

Mine Cicek

Lauren Decker

Jasmin Divers

Ray Ebert

Tonya Farris

Valerie Flaherman

Thomas Flotte

Andrea Foulkes

Rachel Gross

Sally Hodder

Leora Horwitz

Beth Karlson

Stuart Katz

Craig Lefebvre

Lei Lei

Shawn Murphy

Lisa Newman

Michelle Olive

Tony Punturieri

Lisa Schwartz Longacre

Upinder Singh

Stephen Thibodeau

Andrea Troxel

David Warburton

Jordan Weyer

**Health Equity/ PRO / Community Engagement**

Brett Anderson

Sujata Bardhan

Leah Castro-Baucom

Deena Chisolm

Claudia Corchado

April Joy Damian

Casey Daniel

Soham DasGupta

Walter Dehority

Candace Feldman

Josh Fessel

Lisa Goldman Rosas

Carol Horowitz

Janice John

Dhruv Khullar

Keila Lopez

Karen Lutrick

Carina Marquez

Shelly McDonald Pinkett

Larissa Myaskovsky

Lidia Regino

Kim Rhoads

Gelise St John Thomas

Sarah Stewart de Ramirez

Joel Tsevat

Carlos Valencia

Nita Vangeepuram

Anita Walden

Zanthia Wiley

Neely Williams

Shonna Yin

**Immunology & Hematology**

Hulya Bukulmez

Chris Chute

Karen Costenbader

Betty Diamond

Rao Divi

Nahed El Kassar

Nathan Erdmann

Frances Eun-Hyung Lee

Alicia Gaffney

Sacha Gnjatic

Jason Goldman

Timothy Gondre-Lewis

Jim Heath

Jennifer Hossain

Chao Jiang

Ellen Kraig

Joy Liu

Aprajita Mattoo

Joshua Milner

Sindhu Mohandas

Janko Nikolich-Zugich

Princess Ogbogu

Michael Peluso

Bellur Prabhakar

Jay Raval

Marian Sullivan

Paul Thuluvath

PJ Utz

Sidney Whiteheart

**Integrative Physiology**

Nina Caplin

Dawood Darbar

Steven Deeks

Katie Fain

Aloke Finn

Thomas Flotte

David Goldstein

Meredith Hay

Ellie Hirshberg

Charles Howell

Barbara Karp

Dean Kellogg

Rebecca Letts

Meisha Mandal

Janet Mullington

Asa Oxner

David Putrino

Jacqueline Rutter

Joel Trinity

John Wood

Roham Zamanian

**Mechanistic Pathways**

Christian Bime

Steven Bradfute

Benjamin Chen

Tom Connors

Krista Coombs

Glenn Fishman

Maria Gennaro

Timothy Henrich

Prasanna Jagganathan

Judith James

Boris Juelg

Christina Kim

Sindhu Mohandas

Michael Portman

Brian Reeves

Jalees Rehman

Ignacio Sanz

**Metabolic Disorders**

Leyna Aragon

Irina Buhimschi

Floyd (Ski)

Ralph DeFronzo

Emily Gallagher

Jennifer Hossain

Mandana Khalili

Angesom Kibreab

Tracey McLaughlin

Nandini Nair

Venkat Narayan

Elizabeth Phillips

Jane Reusch

Ivonne Schulman

Aasma Shaukat

Deborah Wexler

Jonah Zaretsky

**Microbiology**

Bill Alexander

Ami Bhatt

Hassan Brim

Shari Brosnahan

John Coffin

Adolfo Garcia-Sastre

Maria Gennaro

Joerg Graf

Timothy Henrich

Hye-Sook Him

Nahed Ismail

Jeanne Marrazzo

Rebecca McGrath

Sindhu Mohandas

Christopher Montgomery

Radu Postelincu

Ryan Ranallo

Adam Spivak

Mehul Suthar

Mary Thomas

Guangming Zhong

**National Community Engagement Group**

Teresa Akintonwa

Jasmine Briscoe

Heather Elizabeth Brown

Megan Carmilani

Marta Cerda

Debra Copeland

Felicia Davis

Roberto Garcia

Nick Guthe

Yvonka Hall

Kevin Kondo

Fadwa Lawrence

Lydia Lerma

Jacqui Lindsay

Christine Maughan

Thomas (Tony) Minor

Marjorie Roberts

Nitza Rochez

Brittany Taylor

Susanna Tranguch

Hyatt Vincent

Heather Yates

**Neuropsychiatric**

John Andrefsky

Bryan Bander

Douglas Bremner

Michael Carrithers

Melissa Cortez

Richard Gallagher

Alejandra Gonzalez

Joanna Hellmuth

Barbara Karp

Tammy Kershner

Shawn Murphy

Ganesh Murthy

Sharon H. O'Neil

Lisa Prentiss

Caitlin Rollins

Jonathan Rosand

Scott Russo

Amy Salisbury

Alan Seifert

Sudha Seshadri

Eyal Shemesh

Wendy Silver

Naomi Simon

Leanne Williams

**Omics**

Masanori Aikawa

Hassan Ashktorab

Noam Beckmann

Mike Enger

Joaquin Espinosa

Xiaowu Gai

Stephen Hewitt

Benjamin Horne

Jessica Lasky-Su

Cheryl Maier

Meisha Mandal

Lucio Miele

Emmanuel Mongodin

Lauren Nichols

Nadia Roan

Mark Russell

George Saade

Kumar Sharma

Stephanie Shiau

Jun Sun

Stephen Thibodeau

Sam Yang

**Participant Experience**

Nina Blachman

Natalie Boutin

Phoebe Burton

Marina Catallozzi

Cheryl Clark

Beth Dworetzky

Belinda Edwards

Robert L. Ferrer

Beatrice Huang

Suzanne Judd

Sarah Laury

Hugh Musick

Divya Pathak

Kristen Pogreba-Brown

Hengameh Raissy

Lynne Richardson

Russell Rothman

Laura Wagner

Ann Wallace

**Population Science**

Paul Barach

Melissa Bondy

Victor Castro

Mine Cicek

Joanne Elena

Kacey Ernst

Josh Fessel

Dan Fort

Brian Hendricks

Bertha Hidalgo

Cory Hussain

Carmen Isasi

Dan Kelly

Adeyinka Laiyemo

Margaret Lanca

Juan Lewis

Beth Linas

Heidi May

Kimberly McHugh

Naoko Muramatsu

Girish Nadkarni

Susan Nance

Kian Nguyen

Priscilla Pemu

Lisa Postow

Suchitra Rao

Dimpy Shah

Sidd Shenoy

Stephanie Wilson

Dana Wolff-Hughes

**Presentations and Publications Oversight**

Ingrid Bassett

Diana Berrent

Andra Blomkalns

Hassan Brim

Rebecca Clifton

Nathan Erdmann

Kristine Erlandson

Josh Fessel

Valerie Flaherman

Margot Gage

Mark Goldberg

Edmond Kabagembe

Tammy Kershner

Patricia Kinser

Jonathan Klein

Gregory Laynor

Grace Lee

Grace McComsey

Brian McCrindle

Julie McMurry

Girish Nadkarni

Priscilla Pemu

Dustin Rabideau

Erika Rosenzweig

David Warburton

**QA/QC Data Integrity**

Audie Atienza

Charlie Bailey

James Chan

Mine Cicek

Hannah Davis

Kathi Diviak

Ray Ebert

Dan Fort

Jennifer Gander

Janos Hajagos

Kellie Hawkins

Shahidul Islam

Dan Kelly

Tammy Kershner

Patricia Kovatch

Michelle Lamendola-Essel

Simon Li

Daniel Liu

Holden Maecker

Emily Pfaff

Anisha Sekar

Zaki Sherif

Vignesh Subbian

Anand Viswanathan

Jennifer Wheeler

Meredith Zozus

**Study Design**

Yvette Burgos

Chris Chute

Katharine Clouser

Juan Espinoza

Megan Fitzgerald

Valerie Flaherman

Elizabeth Karlson

Barbara Karp

Lawrence Kleinman

Adeyinka Laiyemo

Emily Levitan

Gabrielle Maranga

Gailen Marshall

Jeffrey Martin

Robin Mermelstein

Torri Metz

Sindu Mohandas

Jennifer Muszynski

Jane Newburger

Igho Ofotokun

Sairam Parthasarathy

Kyung Rhee

Lumy Sawaki-Adams

Mary Beth Scholand

Howard Sesso

Nora Singer

Jessica Snowden

Cheryl Stein

Lauren Stiles

Melissa Stockwell

Kelan Tantisira

Barbara Taylor

Tanayott Thaweethai

Juan Wisnivesky
