# Supplementary material for: Researching COVID to Enhance Recovery (RECOVER) adult study protocol: Rationale, objectives, and design
Source: PLoS One. 2023 Jun 23;18(6):e0286297. doi: 10.1371/journal.pone.0286297 (PMC10289397; doi:10.1371/journal.pone.0286297)
Supplement: S1 File — (PDF) [file pone.0286297.s012.pdf]

[Insert logo here]

Level 3 Research Participant Informed Consent Form

**Title of Study:**	**NIH RECOVER: A Multi-site Observational Study of Post-Acute Sequelae of SARS-CoV-2 Infection in Adults**<br>Study Number: S21-01226
**Site Study Leader:**	Name of the Principal Investigator<br>Department of Principal Investigator<br>Applicable Medical Center<br>Address<br>Phone Numbers
**Study Leaders:**	**Stuart Katz, MD MS**<br>NYU Grossman School of Medicine<br>Leon H. Charney Division of Cardiology<br>530 First Avenue, Skirball 9R<br>New York, NY 10016<br><br>**Leora Horwitz, MD, MHS**<br>NYU Grossman School of Medicine<br>Department of Population Health<br>227 E 30th St, #633<br>New York, NY 10016<br><br>**Andrea Troxel, ScD**<br>NYU Grossman School of Medicine<br>Department of Population Health<br>180 Madison Ave, 5-55<br>New York, NY 10016
**For questions or concerns about the study, please call:**	Clinical Science Core Contact Information:<br>**Stuart Katz, MD MS**<br>NYU Grossman School of Medicine<br>Leon H. Charney Division of Cardiology<br>530 First Avenue, Skirball 9R<br>New York, NY 10016<br>646-501-0119
**Emergency Contact:**	Insert Emergency Contact<br>Insert Phone Number/Pager, etc

1. About volunteering for this research study

You are being invited to participate in Level 3 of the overall research study, "**A Multi-site Observational Study: NIH RECOVER Study of Long COVID in Adults**" that you are already participating in. You can choose whether or not you want to take part in this part of the study.

Before you decide if you want to be a part of this study, you will need to know what the study is about, what you will be asked to do, and the possible risks and benefits. You may also want to talk about this study and this form with your family, friends, or doctor. If you have any questions about the study or about this form, please ask us. If you decide to be in this part of this study, you must sign this form. We will give you a copy of this form to keep once you have signed it.

If you are getting this form, it means we would like you to get Level 3 testing.

2. Why are we doing this study?

The reason we are doing more tests on some people in the overall study is to better understand exactly how people's organs are affected in long COVID. We have already gotten a lot of information from you about your symptoms, test results and how different parts of your body are working. The reason for doing this part of the study is to look even more carefully at how your body is working so that we can learn more about long COVID.

You may have been selected to do some of these tests because you still have symptoms of COVID and we need to understand why you have these symptoms. Or, you may have been asked to complete a level 3 test even if you do not have symptoms. We randomly selected (like a flip of a coin) some people without symptoms to have level 3 tests so that we can compare results to those with symptoms.

3. How long will I be in the study? How many other people will be in the study?

You are participating in the overall study for up to four years. However, you will only get each Level 3 test once. We expect that about one in five people (20 percent) who are in the main study will be asked to get each of the Level 3 tests.

4. What will I be asked to do in the study?

Table 1 shows some of the tests you may be asked to do, why you are being asked to do them and where you go to have them done. Licensed clinicians or technicians will do all the tests. You can still stay in the study even if you do not agree to all the Level 3 tests we ask you to do.

Some of the procedures below will also require you to complete a clinical consent form. If you get any of these procedures you will be asked to sign a separate clinical consent, which will discuss the risks in more detail.

Table 1: Level 3 tests and procedures, description of the test and what it is used for.

Location	Name of test	What will happen	What it is for
Office	Nerve conduction study (NCS) (test of the	You will have small electrodes taped to your skin that will make tiny electric	To check for a problem

Approved For Period: 2/3/2022 - 9/16/2022

Location	Name of test	What will happen	What it is for
	nerves)	shocks to measure how your nerves are working. There are no needles and nothing will poke into your skin.	with the nerves or muscles
Office	Electromyography (EMG) (test of the muscles)	Thin needles (about the width of a hair) are put into your muscles to measure whether your muscles are responding the right way to your nerves. The electrical activity in your muscle is recorded onto the EMG machine. The muscle is tested while you are resting and while you are moving the muscle.	
Office	Skin biopsy (small piece of skin taken from the body)	You will have a small amount of medicine put into your skin using a needle to numb it (local anesthesia), usually on the lower leg. Then a tool about the size of a pen will be used to take a small piece of your skin, about the size of a thin straw. The doctor will close the wound with a bandage. No stitches are needed. This takes about 20 minutes. Pregnant women cannot do this test.	
Office	Muscle biopsy (small piece of muscle taken from the body)	You will have a small amount of medicine put into your skin using a needle to numb it (local anesthesia).A small cut (less than a half inch) is then made into the skin. A needle is then put into the muscle and a small piece of muscle remains in the needle when it is taken out of the body. This is about the size of 2 to 3 grains of rice. The needle may need to be put into the muscle more than one time to get a large enough piece for us to look at.<br><br>The muscle we will take a piece of is the outer thigh muscle, about mid-way between the knee and hip. If we cannot use the thigh, we will take a piece from the deltoid muscle in the arm. Pregnant women cannot do this test.	To check for problems in the muscles
Office	Lumbar puncture (spinal tap)	The lumbar puncture will be done while you are lying down on your side on a table or when you are sitting. You will have a small amount of medicine put into the skin with a needle to numb it (local anesthesia) over the lower back. When the area is numb, a very thin needle will	To check for damage, infection, or inflammation (irritation) in the brain

Location	Name of test	What will happen	What it is for
		be put into the spinal canal in the lower back well below the end of the spinal cord. About 25 mL (1 2/3 tablespoons) of spinal fluid will be collected for testing and storage. This test usually takes between 30 to 60 minutes. After the lumbar puncture, you will stay resting in bed for up to 1 hour. You should not do any exercise or do a lot of physical activity for 24 hours after the lumbar puncture. This includes jogging, bike riding, lifting, bending, doing housework, and gardening. Pregnant women cannot do this test.	
Office	Tilt table test and cardiovagal testing	We will test how your body acts based on the way your body is moved. You will lie on a table that tilts. We will tilt the table to move you into different positions (lying down, close to standing). While we do this, we will look at how fast your heart is beating and your blood pressure during the test. This test usually takes 30 minutes. Pregnant women cannot do the tilt table part of the test.	To find out why there low blood pressure or fainting
Office	MRI of the heart or brain	This test uses strong magnets and radio waves to take a picture of your brain or of your heart. You will be asked to lie down on a table. The table will move you inside the machine, which looks like a long tube. You will be asked to stay still while the pictures are being taken. This test takes about one hour. We may give you an injections of a chemical that will improve the quality of the picture. Pregnant women can do this test but will not be given the chemical.	To look for a problem in the brain or the heart
Office	Bronchoscopy (test to look at the air passage ways in the lungs)	You will be given some liquid numbing medicine to gargle with, and then some numbing medicine to breathe in through a nebulizer machine. We will also numb the opening of your nose with some numbing jelly. We will use a needle to put a tiny tube into a blood vessel (called an IV) and will give you medicine through it to make you drowsy and calm. A tube with a camera at the end will be placed through the opening of your nose into the lungs.	To check for problems in the lungs

Location	Name of test	What will happen	What it is for
		Using the tube, we will wash some fluid through each part of the lungs and take it so we can see what is inside the lungs. We will also rub the airways lightly with a brush to collect cells from the airways. After the test, you will need to stay in the office for 2 hours so that we can watch you and make sure you are ready to go home. Pregnant women cannot do this test.	
Office	Right heart catheterization (test to see how well the heart is working)	You will have a small amount of numbing medicine put into your skin with a needle (local anesthesia) in the groin, arm, or neck. We will also use a needle to put a tiny tube into your blood vessel (called an IV), and will give you medicine through it to make you sleepy and relaxed. A catheter (a long hollow tube) is placed through a needle into your vein and passed to your heart while using special X-rays (fluoroscopy). We will record pressures and oxygen level measurements in the heart and lungs. Depending on the problem, we may do more measurements during exercise or after you breathe a medicine. At the end of the test the catheter is taken out, and pressure is put on the area where the catheter went into the skin to keep it from bleeding. Pregnant women cannot do this test.	To check how well your heart is pumping and to measure the blood pressure in your heart and the main blood vessels in your lungs.
Radiology	Cardiac imaging with meta-iodobenzylguanidine (mIBG) (test of the heart)	We will use a needle to put a tiny tube into your blood vessel (called an IV). We will then put a radioactive material called Meta-iodobenzylguanidine or MIBG into the IV. About 15 minutes later, we will take a picture of your heart with a special machine that can see the chemical. We will take another picture 3 hours later. Pregnant women cannot do this test.	
Office	Gastric emptying study (test of how your stomach is working)	You will eat some food, usually an egg, that has had a radioactive material added to it. After you eat it, you will be placed under a scanner and the food will be put under a machine where we will be able to see the food as it moves out of the stomach and into the small intestine. This	To check how well the top part of the digestive system is working and to check for

Location	Name of test	What will happen	What it is for
		scan could take a few hours, depending on how long it takes for the food to empty from the stomach. The radioactive material is not absorbed by the body or dangerous to yourself or others. Pregnant woman cannot do this test.	any problems
Office	Upper endoscopy (test to look at the top part of your digestive system)	You will be given some liquid numbing medicine to gargle with. Then a small tube with a camera and tools will be placed in the mouth and into the stomach and upper intestines. We will look at the stomach and intestines, and if anything looks abnormal, take a small piece of it. Pregnant women cannot do this test.	
Office	Colonoscopy with or without biopsy (test to look at the bottom part of your digestive system)	You will drink some fluid the day before the test that will give you diarrhea and clean out your digestive system. You will get some medicine to make you sleepy, and then a tube with a camera on the end is put in the rectum and moved through the large intestine, known as the colon. Small pieces may be taken from the body, like growths or polyps and biopsies of the colon lining. Pregnant women cannot do this test.	To check the lower part of the digestive system problems

Future use of information found from tests

Information that identifies you, such as name and address, will be removed from the identifiable results. After this information is removed, information and/or results from the tests may be used for future research studies or shared with other researchers and we will not ask for additional informed consent from you to use this information.

5. What are the possible risks or discomforts?

Being in this study may involve some risks or discomforts from study procedures. In addition to the risks listed below, there may be risks that have not been seen before. You should contact the Study Site Leader if you are worried about anything while you are a part of the study.

Numbing medicine

Many of the tests in this form use numbing medicine (lidocaine) to reduce pain during the test (including the nerve conduction study, electromyography, skin and muscle biopsies, lumbar puncture, and right heart catheterization). The numbing medicine can cause redness or swelling in some people. Some people are allergic to the numbing medicine or to the things used in the tests. Symptoms of any allergic reaction can include a rash, hives, itching, and/or difficulty breathing, closing of the throat, swelling of the lips, tongue or face, and rarely death. It is important to tell the study doctor of any allergies you may have so we can reduce this risk. There are trained medical people and emergency

Approved For Period: 2/3/2022 - 9/16/2022

equipment and medicines available in the places where the tests are performed to treat you if you have an allergic reaction.

Nerve conduction study (NCS) and electromyography (EMG)

The nerve conduction study can be uncomfortable because the test involves putting small electrodes on your skin that produce a tiny electrical shock. Most people experience this as a pinch or tingling sensation. These shocks cannot cause permanent injury or damage; however, they can interfere with any electrical devices you have in your body, such as a pacemaker, defibrillator, cochlear implant or spinal cord stimulator. If you have one of these devices, you should tell the doctor doing the study.

The electromyography (EMG) can also be uncomfortable because it involves putting a skinny disposable needle (about the width of a hair) into the muscles being tested. Since every patient tolerates discomfort and pain differently, it is impossible to predict how discomforting and/or painful this test will be for you. Most patients report discomfort as mild. Minimal bleeding and, rarely, bruising may occur during or after the test. Therefore, it is very important to tell us if you have a bleeding problem, or are taking blood thinning medications such as warfarin or heparin. After the test, the muscle (or several muscles) may feel tender and bruised for a few days. The needles we use are sterile but could possibly become infected, although the risk is minimal.

Skin and muscle biopsies

Risks of the skin or muscle biopsy include bleeding, soreness at the place where you had the pieces taken, bruising or (rarely) infection. Less than 1 out of 100 people have a lot of bleeding or an infection from a skin biopsy. To reduce the risk of infection, we will ask you not to get the area wet for 24 hours. Rarely, people may get a small scar where the skin was cut. We will not do this test on pregnant people. Please tell the study staff if you are pregnant or plan to be pregnant.

Lumbar puncture (spinal tap)

Less than 3 out of 100 people may have a headache lasting 1-2 days after the test. This can be treated with Tylenol, fluids or caffeinated drinks. Rarely, headaches may be more severe and last as long as a week. Very rarely, severe headaches may be treated by using a blood patch. This is where a small amount of your own blood is put into the place you had the lumbar puncture. This is done by a doctor called an anesthesiologist and it will immediately stop the headache.

There is a slight risk of infection where we poke the needle in for the lumbar puncture. Although very rare, it is possible that you may have an allergic reaction to the numbing medicine (lidocaine 1%) used to put the needle in for the lumbar puncture, or other things used as part of the test. This would cause swelling and a rash on your skin where the numbing medicine was put in. Please tell us if you have ever had a problem with numbing medicine before (such as when you were visiting the dentist). As with any drug, there may be side effects that we do not expect.

There is a small risk of bleeding, which we lower by thoroughly checking your bloodwork and medication list and not doing the test if you are at any risk of bleeding. In very rare cases bleeding in the spinal canal can put pressure on the nerves or even the spinal cord. In these extremely rare cases, the blood clot may need to be taken out in a surgery. Having a lumbar puncture can be risky in people with an infection around the brain, like meningitis, or in those with a brain tumor. You will not have a lumbar puncture if your examination or brain MRI scan shows a condition that makes having a lumbar puncture more risky. We will not do this test on pregnant people. Please tell the study staff if you are pregnant or plan to be pregnant.

Approved For Period: 2/3/2022 - 9/16/2022

Bronchoscopy

The main risk of this procedure is fever, which occurs in 5 out of 100 people (5 percent). This can be treated with acetaminophen (Tylenol) and usually does not last more than a day. Pneumonia occurs rarely. Even more rarely, part of the lung can collapse. We will check for this with an X ray after the test is done. To reduce risk, we will not do this test on people with severe lung disease. Using too much numbing medicine during the test can cause harm including seizures or even death. To avoid this we place strict limits on the amount that can be used. The sedation medicine you will get to keep you relaxed during the test may lower your blood pressure or your breathing rate, or make you woozy or confused for a while after the test. These can be treated with fluids or oxygen, or by giving you less medicine. We will not do this test on pregnant people. Please tell the study staff if you are pregnant or plan to be pregnant.

Radiation Exposure

Your participation in this study will involve exposure to radiation from nuclear medicine (cardiac imaging with MIBG, and a gastric emptying scan) and radiologic procedures (lumbar puncture and right heart catheterization). This exposure is not necessary for your medical care, is for research purposes only and is necessary to obtain the desired medical information.

Radiation has been shown to cause cancer from exposures that are higher than the additional annual radiation dose you will receive by participating in this study. According to the International Commission on Radiological Protection (ICRP), the increased risk of health effects, such as cancer, from radiation doses of this amount is either too small to be observed or nonexistent. The effective radiation dose you will receive from these research scans is approximately 17 mSv. The total effective dose you will receive from all combined research scans is approximately 39 mSv. The organ receiving the highest dose in this study is the heart. The effective dose you will receive from your participation is comparable to 13 times the yearly dose from natural environmental radiation in the US (3.1 mSv) and within the limits of 50 mSV, which is set by the FDA for individuals participating in basic research studies.

Please inform your researcher if you have been exposed to radiation as a result of any other research studies or part of your clinical care. If you participate in future studies that involve the use of radiation, you should discuss it with the researchers performing those studies.

Pregnant Participants:

Pregnant women cannot be exposed to radiation. Women of child bearing potential must have a negative pregnancy test before they can participate. It is important to understand that a negative pregnancy test may occur even if you are pregnant depending on the timing of the test relative to ovulation.

Tilt table test and cardiovagal testing

You may feel lightheaded or weak or may faint while you are tilted or when holding your breath. If you have symptoms related to the tilting, or if your blood pressure or heart rate changes too much, we will bring you back to the lying position right away. We will continue to monitor you until any symptoms improve and your blood pressure and heart rate are back to what is normal for you. Pregnant women cannot do the tilt table test.

MRI

MRI uses strong magnetic fields and radio waves to make images of the inside of your body. MRI does NOT involve high-energy radiation (like x-rays). For most people MRI is very safe. However, if you have anything made of metal on your skin or inside your body, MRI may not be safe for you, and you must tell study personnel before your scan. Also, if you have any electronic devices on the

outside or inside of your body, you must tell study personnel about those too. Some things, like tattoos, may have metal materials in them even though you might not realize it. For this reason, study personnel will give you a checklist of things that have metal or electronic parts in them. You must read the list carefully before your scan and put a checkmark next to everything that applies to you.

The following paragraphs will describe the possible risks of MRI. To reduce many of these risks, you will be given an emergency squeeze ball to hold in your hand during the scan. If you feel any discomfort, you should squeeze the ball. This sets off an alarm that the technologist can hear. The technologist will then talk to you and will stop the scan if you want. There is a microphone in the scanner so that you can communicate with the technologist. However, the scanner makes a lot of noise when it is running, and the technologist may not always hear what you say. If you need to get the technologist's attention, you should squeeze the ball. Remember, if at any point you feel uncomfortable and want to stop the scan, just squeeze the ball and tell the technologist.

Risks from metal
The strong magnetic field in the scanner will pull on things that contain certain types of metal. If someone takes a metal object into the scan room, it might fly towards the scanner and hurt you. For this reason, everyone (including you) must remove everything metal from their clothes and pockets before going into the scan room. Also, the door to the scan room will be kept closed during the scan to prevent unauthorized people from walking in.
If you have something metal inside your body, the scanner might pull on it and make it move. You must tell study personnel before your scan if you have anything metal inside your body.
Some types of metal might heat up when the scanner is running. If you feel any burning sensation during the scan, you should squeeze the emergency ball and the technologist will stop the scan.

Risks from electronic devices
If you have any electronic devices on the inside or outside of your body, the scanner might make them stop working properly. For this reason, you must tell study personnel before your scan if you have anything electronic on or in your body.

Burns
Metal is not the only thing that can cause burns in MRI. It's possible (although very rare) to get burned by touching the inside walls of the scanner or by making skin-to-skin contact. The technologist will give you a blanket or cushions so that you don't touch the inside walls of the scanner. You should also avoid letting your hands or legs touch each other. Remember, if you feel any burning sensation during the scan, you should squeeze the emergency ball and the technologist will stop the scan.

Tinnitus (ringing in the ears) and hearing loss
The scanner makes very loud sounds while it is running. You will be given earplugs or headphones to wear during the scan. Make sure you roll the earplugs tightly and let them expand in your ears so that they work properly. If the sound of the scanner is still so loud that it causes you discomfort, squeeze the emergency ball and tell the technologist. This is important because very loud sounds can cause ringing in the ears or even hearing loss.

Feeling warm or hot
The radio waves used in MRI are like those your cellphone uses, but much stronger. Sometimes they are strong enough to make you feel warm (just like standing in bright sunshine makes you feel

Approved For Period: 2/3/2022 - 9/16/2022

warm). MRI scanners are designed to try to avoid you getting too hot. However, if you start to feel uncomfortable, squeeze the emergency ball and tell the technologist.

Peripheral nerve stimulation (tingling or twitching)
The magnetic field inside the scanner changes very quickly while the scanner is running. If it changes too quickly, it can give you tingling sensations or make you twitch. MRI scanners are designed to try to avoid this. However, if you experience tingling or twitching, squeeze the emergency ball and tell the technologist.

Claustrophobia (discomfort in enclosed spaces)
Some people get panic attacks inside enclosed spaces. This is called ‘claustrophobia’, which means ‘fear of confined spaces’. If you know that you are claustrophobic, tell study personnel before your scan. Some people only find out they are claustrophobic when they have an MRI for the first time. If you feel anxious or panicky inside the scanner, squeeze the emergency ball and the technologist will get you out.

Quench
In very rare circumstances, the scanner can lose its magnetic field. This happens very suddenly and is known as a ‘quench’. The helium that helps keep the magnetic field strong will then escape from the scanner. The scanner is connected to a vent so that the helium will go outside the building. However, if for some reason the vent doesn’t work properly, helium might fill the scan room, making it difficult to breathe. In the very unlikely event of a quench, the technologists will get you out of the scanner immediately.

Right heart catheterization
While placing the catheter in the vein, a bruise may occur at the place where the needle went in and, in rare cases, bleeding, infection or blockage of the vein may also occur. If the catheter is put in a vein in the neck, there is a very low risk of lung collapse. In this case, you may be admitted to the hospital to have a tube placed in the chest to fix the lung collapse. It is possible that the test will cause abnormal heart rhythms, which are felt as the heart beating fast (palpitations). In very rare cases, we may need to give you a special medicine or treatment. Serious changes may mean you may need to have emergency defibrillation (giving an electrical shock to the heart). The sedation medicine you will get to keep you relaxed during the test may lower your blood pressure or your breathing rate, or make you woozy or confused for a while after the test. These can be treated with fluids or oxygen, or by giving you less medicine. We will not do this test on pregnant people. Please tell the study staff if you are pregnant or plan to be pregnant.

Cardiac imaging with meta-iodobenzylguanidine (MIBG)
MIBG is an FDA-approved radioactive material, which means that it will expose you to a small amount of radiation, equivalent to about 15 months of average yearly radiation exposure from the environment. It may also cause your blood pressure to increase. You may be asked to stay at the place where the test is done for about 30 minutes to 1 hour after the test to watch over your blood pressure. We will not do this test on pregnant people. Please tell the study staff if you are pregnant or plan to be pregnant.

Gastric emptying study
This test may cause diarrhea or make you light-headed. Please contact the study director if your diarrhea keeps going for 48 hours. We will not do this test on pregnant people. Please tell the study staff if you are pregnant or plan to be pregnant.

Upper endoscopy

The procedure may rarely cause bleeding from where pieces are taken or cause a hole in the esophagus, stomach or small intestine that may need surgery to repair. The sedation medicine you will get to keep you relaxed during the test may lower your blood pressure or your breathing rate, or make you woozy or confused for a while after the test. These can be treated with fluids or oxygen, or by giving you less medicine. We will not do this test on pregnant people. Please tell the study staff if you are pregnant or plan to be pregnant.

Colonoscopy

The colonoscopy is safe and very rarely may cause a hole in, or tear, of the colon that would need to be treated with medicine (antibiotics), lead you to stay in the hospital and possibly need surgery to fix the hole or tear. This risk is slightly higher if you have a biopsy (piece of tissue removed) during the test. You may pass gas or have bloody stools. The sedation medicine you will get to keep you relaxed during the test may lower your blood pressure or your breathing rate, or make you woozy or confused for a while after the test. These can be treated with fluids or oxygen, or by giving you less medicine. You may have nausea, vomiting, bloating or belly pain while drinking the liquid to clear out your intestine. Serious side effects are rare. We will not do this test on pregnant people. Please tell the study staff if you are pregnant or plan to be pregnant.

Below, we list which of these tests you are being asked to do at this time. Please put your initials in the “agree” or “do not agree” section to show if you agree to do the test.

Level 3 test	Picked for you	Agree	Do not agree
Nerve conduction study (NCS)			
Electromyography (EMG)			
Skin biopsy			
Muscle biopsy			
Lumbar puncture or spinal tap			
Tilt table test			
Brain MRI, with and without gadolinium contrast			
Bronchoscopy			
Cardiac MRI, with and without gadolinium contrast			
Right heart catheterization			
Cardiac imaging with meta-iodobenzylguanidine (mIBG)			
Gastric emptying study			
Upper endoscopy			
Colonoscopy			

6. Can I be in the study if I am pregnant or breastfeeding?

You can be in the study if you are pregnant or breastfeeding, but you will not undergo the following procedures: tilt table test, bronchoscopy, MRI with contrast, colonoscopy, lumbar puncture, muscle biopsy, right heart catheterization, cardiac imaging with mIBG, skin biopsy, or upper endoscopy.

7. What if new information becomes available?

During this study we may find information that could be important to you. This includes information that might cause you to change your mind about being in the study. We will let you know as soon as possible if this kind of information is found.

Findings of research blood tests and research images from scans

In this part of the form, we are asking you to let us know if you would like to know about any results we find in the research laboratory.

You will be able to see the results of the tests done by a certified clinical laboratory in your health record. At some places, your main doctor, and other people who care for you and who can look at your health records will be able to see this information. Also, each of the clinical laboratory tests and scans done as part of this study will be looked at by an expert, who may find something that is not normal. If something that could affect your health is found, someone from the study team will talk to you in person or by phone about this new information.

Some tests may be done in a research laboratory. A research laboratory does tests where the results are just used for research; the results may not be reliable for use in your health care, or we may not know what the results mean for your health. Research laboratory tests may be done while you are in the study, or in the future after the study is finished. The law does not let us give you results of tests done in research laboratories because they are not meant for patient care.

8. What are the possible good things (benefits) from being part of the study?

Being part of the study may help you and your doctor better understand problems that are due to COVID. The results of the study will be important in helping patients, caregivers, and parents understand how COVID affects the body’s long-term reaction to COVID.

9. What other choices do I have if I am not part of the study?

This study does not provide treatment and the tests are being done for research purposes, not to provide you with health care. If you have COVID symptoms, you will have to be treated outside the study by your own doctors. If you do not take part in this study, you can get any tests you need for COVID care from your own doctors. Deciding not to be part of the study will not affect your health care now or in the future, how you pay for health care or if you can get health insurance.

10. Will I be paid for being in this study?

We will pay you back for travel costs to and from the study site and any hotel costs related to the study. In order to be paid, you must give the receipts to the study staff.

Approved For Period: 2/3/2022 - 9/16/2022

Because you will be getting money to be part of this research study, you will need to give the study staff either your Social Security number or your Alien Registration number. You will also be asked to fill out a form called the IRS W9. This is because [study site] is required by laws of the United States to report to the Internal Revenue Service (IRS) any amounts that are paid to research participants that are equal to or greater than $600. You may need to pay taxes on payments for research that are greater than $600. If you do not have either of these numbers or are not willing to fill out the IRS W9 form, you may be in the study but will not get any money.

You must keep a record of all the money given to you for being part of this research study and any other research study for each year (from January to December) that you are part of research studies. You must let us know right away when the total amount of money you get for being part of research studies is the same as or likely to be greater than $600 total (not including money to pay for the cost of travel) in any one year (from January to December).

The use of your information and study samples may lead to new tests or drugs, or other things that may be sold to make money. A patent or license may be gotten for these things to keep other people from making, using, or selling these things. There are no plans to give any money to you if this was to happen.

11. Will I have to pay for anything?

There are no costs to you related to being part of the study. The NIH will cover the costs of your being part of the study. You or your insurance company will not be asked to pay for the costs of your visits related to the study. You and/or your health insurance may be billed for the costs of health care during this study if these costs would have happened even if you were not in the study. If your insurance does not cover these costs or you do not have insurance, these costs will be up to you to take care of.

12. What happens if I am injured from being in the study?

For emergencies, call 911. If you think you have been hurt because of being part of this research study, tell the Study Site Leader as soon as you can. The Study Site Leader's name and phone number are listed at the top of page 1 of this consent form.

If you are hurt because of being part of this research, we will put you in contact with a doctor to give you treatment if you want. We may ask your insurance company, or someone else, if appropriate, to pay for the costs of the treatment due to your being hurt, but you may also need to pay for some of this cost.

There are no plans for the [study site] or NYU Grossman School of Medicine or NYU Langone Health to pay you or give you anything else for being hurt. You do not give up the rights you have under the law by signing this form.

13. When is the study over? Can I leave the study before it ends?

This study will last for 4 years. This study may be stopped early. It is also possible that you may be taken out of the study early for the following reasons:

- The researchers in charge of the study feel it is important to remove you for your health or safety.
- You have not followed study instructions.

- The group funding the study, the main researchers in charge, or people monitoring the safety of the study decide to stop the study.
- More information about Long COVID is known so that the study is no longer needed.

If you do not wish to keep being part of the study, you are free to leave the study at any time. Leaving the study will not affect your care, how your health care is paid for, or what kind of health insurance you can get.

14. How will you protect my confidentiality (privacy)?

Your medical information is protected health information, or “PHI”, and is protected by federal and state laws, such as the Health Insurance Portability and Accountability Act, or HIPAA. This includes information in your research record as well as information in your medical record at NYU Langone Health. In compliance with NYU Langone Health policies and procedures and with HIPAA, only those individuals with a job purpose can access this information.

Medical information created by this research study may become part of your medical record. We may include your research information in your medical record for several reasons, including for the billing of services provided in connection with the study, to securely document any medical services you receive, and so that other members of the NYU Langone Health community who may treat you have access to important information about your health.

You have a right to access information in your medical record. In some cases, when necessary to protect the integrity of the research, you will not be allowed to see or copy certain information relating to the study while the study is in progress, but you will have the right to see and copy the information once the study is over in accordance with NYU Langone Health policies and applicable law.

Certificate of Confidentiality
To help us further protect your confidentiality, this research is covered by a Certificate of Confidentiality from the National Institutes of Health (NIH). The NIH has issued a Certificate of Confidentiality for this research. This adds special protection for the research information (data, documents, or biospecimens) that may identify you.

Research information protected by this Certificate of Confidentiality cannot be disclosed to anyone else who is not connected with the research, without your consent. With this Certificate of Confidentiality, the researchers may not disclose or use research information that may identify you in any federal, state, or local civil, criminal, administrative, legislative, or other action, suit, or proceeding, or be used as evidence, for example, if there is a court subpoena, without your consent. However, disclosure, without your consent, is still necessary if there is a federal, state, or local law that requires disclosure (such as to report child abuse or communicable diseases).

The Certificate of Confidentiality cannot be used to refuse a request for information from appropriate government agencies responsible for project oversight.

Approved For Period: 2/3/2022 - 9/16/2022

The Certificate of Confidentiality does not prevent you from releasing information about yourself and your involvement in this research, including for your medical treatment. Federal regulations may also allow for the use or sharing of information for other scientific research.

By agreeing to be in this research and signing below, you are giving your consent to share research information with others at [study site]. This means that your research information, including lab results, x-rays, MRIs, information about the investigational drug used in this study, may be included in your [study site] electronic medical record.

15. HIPAA Authorization

As noted in the Confidentiality part of this consent form above, laws in the U.S. have been made so that we, and the researchers working with us, health care providers, and the people who care for you protect the privacy of information that identifies you (used to know who you are) and relates to your past, present, and future physical and mental health problems. We are asking for your permission (authorization) to use and share your health information with others related to this study, in other words, in order for this research to happen, including doing and watching over the study.

Your treatment outside of this study, payment for your health care, and your health care benefits will not be affected even if you do not give permission (authorize) the use and sharing of your information for this study.

What information may be used or shared with others related to this study?
All information in your research record for this study may be used and shared with those people who are in the list at the end of this part of the consent form. Also, information in your health record that the research team believes may be important to the study may be looked at by those in the list. This includes, for example, results from your study visits, laboratory tests, body pictures, scans, other tests, surveys, and diaries.

Who may use and share information in connection with this study?
The following individuals may use, share, or get your information for this research study:

- The research team, including the Study Site Leader and other people helping with the study or who are in charge of watching over the study at the place where you signed up to be part of the research study
- The researchers at NYU Grossman School of Medicine, who are in charge of helping with and watching over the study at all the places across the country where the study is happening
- Non-research staff who need this information to do their jobs (such as for treatment, payment (billing), or health care operations)
- The researchers at Massachusetts General Hospital, who are in charge of storing the information for this study, the researchers at Mayo Clinic who are in charge of the research biorepository, and other RECOVER study centers or national centers in charge of storing research information
- The group that funded the study: National Institutes of Health (National Heart Lung and Blood Institute)
- National databases such as the National Center for Biotechnology Information repository or dbGAP
- The ethical review board (also called institutional review board or IRB) that oversees the research and the research quality improvement programs.
- The group that is watching over the safety of patients and families in the study (called the study safety monitoring board). The National Institutes of Health decides who will be in this group.

Approved For Period: 2/3/2022 - 9/16/2022

- A company hired to oversee the quality of the RECOVER research information ((Biomedical Research Alliance of New York)
- People or groups that we hire to do work for the study, such as data storage companies, insurers, and lawyers
- Governmental agencies in charge of watching over or overseeing the research (for example, the US Department of Health and Human Services).
- Health care providers, including your doctors and others who care for you related to this study, and laboratories or other people who are looking at your health information as part of this study.
- Other places that are involved in this research

Your information may be re-disclosed (shared) or used for other reasons if the person who gets your information is not required by law to protect the privacy of the information.

What if I do not want to give permission to use and share my information for this study?
Signing this form is voluntary. You do not have to give us permission to use and share your information, but if you do not, you will not be able to be part of this study.

Can I change my mind and withdraw (take back) permission to use or share my information?
Yes, you may withdraw or take back your permission to use and share your health information at any time for this research study. If you withdraw (take back) your permission, we will not be able to take back information that has already been used or shared with others. To withdraw (take back) your permission, you must write to the Study Site Leader or the Study Leader for the study shown at the top of page 1 of this form. If you withdraw (take back) your permission, you will not be able to stay in this study.

How long may my information be used or shared?
Your permission to use or share your personal health information for this study will never end unless you withdraw it (take it back).

16. The Institutional Review Board (IRB) and how it protects you

The Institutional Review Board (IRB) reviews all research involving people before it can be started and then as long as the study continues. The primary concern of the IRB is for the protection of the people participating in the study. For questions about your rights while participating in the study contact the NYU IRB Office number on (212) 263-4110. The NYU School of Medicine's IRB is made up of doctors, nurses, scientists, and people from the community.

17. Who can I call with questions, or if I am concerned about my rights as a research participant?

You can call the Institutional Review Board (IRB) with your questions or concerns. Our telephone numbers are listed below. Ask questions as often as you want. Stuart Katz, MD is the person in charge of this research study. His name and phone number are listed on the first page of this form. If you want to speak with someone not directly involved in this research study, please contact [insert name of contact or IRB]. You can call them at [insert contact information].

- You can talk to them about:
- Your rights as a research participant
- Your concerns about the research

Approved For Period: 2/3/2022 - 9/16/2022

- A complaint about the research. Also, if you feel pressured to take part in this research study, or to continue with it, they want to know and can help.

A description of this clinical study will be available on http://www.ClinicalTrials.gov, as required by U.S. Law. This website will not include information that can identify you. At most, the website will include a summary of the results. You can search this website site at any time.

When you sign this form, you are agreeing to take part in this research study as described to you. This means that you have read the consent form, your questions have been answered, and you have decided to volunteer.

Name of Participant (Print)	Signature of Participant	Date

Name of Person Obtaining Consent (Print)	Signature of Person Obtaining Consent	Date

Witness to Consent Process for Non-English-Speaking Participants (using a translated consent form OR "Short Form" in Participant's Spoken Language)

<u>Statement of Witness</u>
As someone who understands both English and the language spoken by the participant, I represent that the English version of the consent form was presented orally to the participant in the participant's language and that the participant was allowed to ask questions.

Name of Witness (Print)	Signature of Witness	Date

Witness to Consent of a Participant Who Cannot Read or Write

<u>Statement of Witness</u>
I represent that the consent form was presented orally to the Participant in the participant's language, that the Participant was allowed to ask questions, and that the participant has indicated his/her consent and authorization for participation by (check box that applies).

- ☐ Participant making his/her own "X" above in the participant signature line
- ☐ Participant showed approval for participation in another way; describe:

Name of Witness (Print)	Signature of Witness	Date

Approved For Period: 2/3/2022 - 9/16/2022
