# Supplementary material for: Researching COVID to Enhance Recovery (RECOVER) adult study protocol: Rationale, objectives, and design
Source: PLoS One. 2023 Jun 23;18(6):e0286297. doi: 10.1371/journal.pone.0286297 (PMC10289397; doi:10.1371/journal.pone.0286297)
Supplement: S2 File — (PDF) [file pone.0286297.s013.pdf]

[Insert logo here]

Research Participant
Main Informed Consent Form

**Title of Study:**	**NIH RECOVER: A Multi-site Observational Study of Post-Acute Sequelae of SARS-CoV-2 Infection in Adults**<br>Study Number: S21-01226
**Site Study Leader:**	Name of the Principal Investigator<br>Department of Principal Investigator<br>Applicable Medical Center<br>Address<br>Phone Numbers
**Study Leaders:**	**Stuart Katz, MD MS**<br>NYU Grossman School of Medicine<br>Leon H. Charney Division of Cardiology<br>530 First Avenue, Skirball 9R<br>New York, NY 10016<br><br>**Leora Horwitz, MD, MHS**<br>NYU Grossman School of Medicine<br>Department of Population Health<br>227 E 30th St, #633<br>New York, NY 10016<br><br>**Andrea Troxel, ScD**<br>NYU Grossman School of Medicine<br>Department of Population Health<br>180 Madison Ave, 5-55<br>New York, NY 10016
**For questions or concerns about the study, please call:**	Name of the Principal Investigator<br>Department of Principal Investigator<br>Applicable Medical Center<br>Address<br>Phone Numbers
**Emergency Contact:**	Insert Emergency Contact<br>Insert Phone Number/Pager, etc

1. About volunteering for this research study

You are being invited to take part in a research study. You can choose whether or not you want to take part in this study.

Before you decide if you want to be a part of this study, you will need to know what the study is about, what you will be asked to do, and the possible benefits and risks. You may also want to talk about this study and this form with your family, friends, or doctor. If you have any questions about the study or about this form, please ask us. If you decide to take part in this study, you must sign this form. We will give you a copy of this form to keep once you have signed it.

2. Why are we doing this study?

This study is part of a research project paid for by the National Institutes of Health (NIH) called RECOVER (Researching COVID to Enhance Recovery). We are doing this study to understand how COVID affects the body, and why some people who got COVID are still sick many months after being infected. This condition is called “Long COVID” or “PASC,” which stands for Post-Acute Sequelae of SARS-CoV-2.

The people in this study will be 18 years or older. Both adults who have had COVID and those who have not will be part of the study.

3. How long will I be in the study? How many other people will be in the study?

This study will last up to four years. During that time, you will answer survey questions every three months for up to four years, and will have in-person visits up to three times in the first year and once a year after that. If you get COVID while in the study, you may have extra visits.

We plan to have about 17,680 people in the study from across the United States.

4. What will I be asked to do in the study?

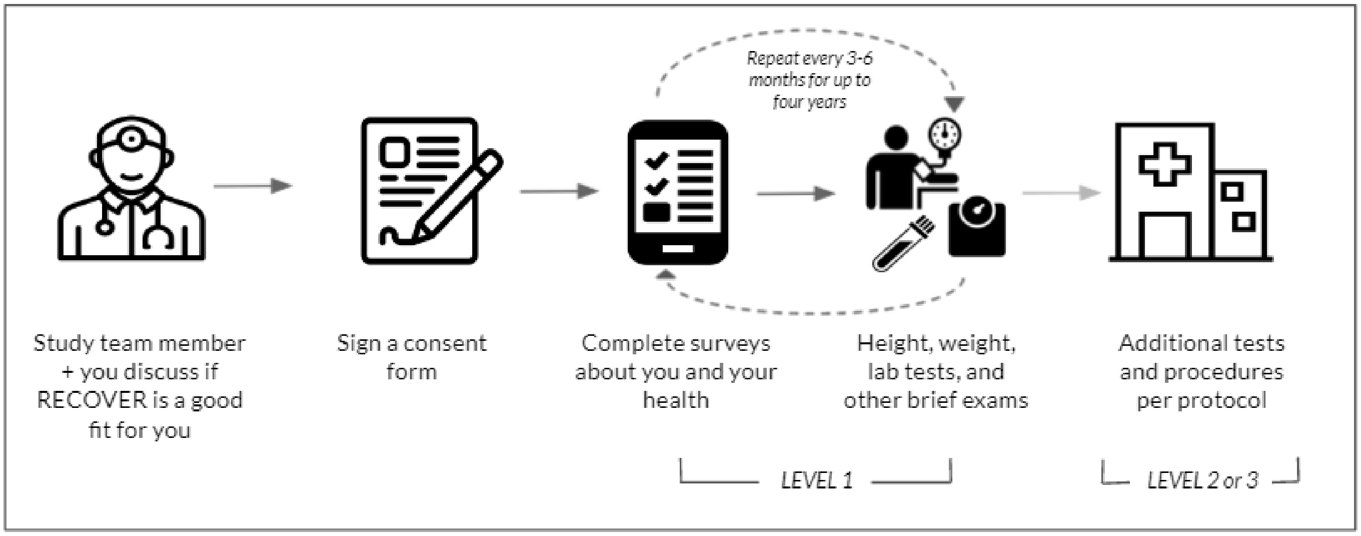

There are three types of study visits: Level 1, Level 2, and Level 3. Everyone who takes part in the study will complete the Level 1 surveys and tests. Only some participants will be asked to do Level 2 or Level 3 surveys and tests. This consent form talks about the surveys and tests for Level 1 and Level 2, and about the surveys and tests for Level 3 that are low risk (very low chance of hurting you). By agreeing to

be a part of this study, you are agreeing to do any of the low risk tests that are appropriate for you. Some Level 3 tests are higher risk, and we will separately ask your permission to do those tests later, if we want you to do them.

If you are pregnant at the time of the study enrollment, or if you were pregnant when you had COVID, we may ask you to enroll your child in the RECOVER children study. Your child's enrollment is not mandatory and will not affect your participation in this study. Not all sites are participating in the children study. If this site is participating in the children study, the investigators from the other study will approach you to see if you would like to enroll your child. In addition, if you were sick with COVID during your pregnancy we will collect medical information about your pregnancy from your medical records.

Level 1 Surveys and Tests

Level 1 Surveys can take place either in person at the study office or at home. When you start the study, the surveys will take about 2 hours. After that, the surveys will take about 45 minutes. The number of questions you will be asked and the amount of time the surveys will take depends on your answers to the questions.

At the first visit, we will ask you questions about you, your health, behaviors, medical problems, and medications. We will also ask some questions about your home, and the neighborhood you live in.
In addition, we will also ask about COVID related items including any testing, COVID vaccine status, and any related health problems you have had. We will look at your hospital records if you had to stay in the hospital because of COVID. If we can't access your health records, we will ask you some of these questions instead.

Every three months after you start the study, we will ask you about how you are currently feeling, and about any new diagnoses (illnesses) your doctors have found or new medicines you are taking.

While you are in the study, we will check national registries (places where information about people across the country is kept) and your medical records to find out about your health and about any tests you have had outside the research study. Your medical records may be included in the study to help the team fully understand your condition.

If you have Long COVID when you start this study, we will let your primary care physician know that you are part of this study. If you join the study while you are still in the hospital, we will reach out to your doctors and nurses to learn about your treatment plans.

Level 1 Tests

At the first visit, we will do the tests listed below and will do them again at 3 and 6 months after you had COVID, if you enroll in the study before that time. If your tests are normal 6 months after infection, we won't do them again. If your tests are not normal, we may do these tests once a year for the rest of the study or until they turn normal.

- *Laboratory blood tests*: We will draw about 3.7 (54 mL) tablespoons of blood from a vein in your arm at each visit, and up to 25.6 tablespoons (378 mL) over 4 years. The blood will be used to check blood sugar levels, cholesterol levels, vitamins, protein levels, hormone levels and other compounds found in the blood

- *Samples for future testing*: We will also take about 5 (71.5mL) more tablespoons of blood at the first visit and up to 21 tablespoons over 4 years to store for testing in the future. We may also collect spit (saliva), poop (stool), liquid from the nose (nasal swab), liquid from the eye (tears), and pee (urine).
- *Exam (Check-up)*: We will measure your weight, height, waist, blood pressure, heart rate, and oxygen level.
- *Monitoring at home between visits*: We may also ask you to use a monitor, like a fitness tracker or an app on a smart phone, computer or tablet. If you do not already have a tracker or device, we may lend one to you to use for the RECOVER study. Some of the devices or apps require you to agree to the company's rules before you can use them. The researchers of the RECOVER study do not control these rules. We will help you understand these rules. The apps or devices will collect information from you and share it with the RECOVER study. Although the RECOVER study will protect the copy of your information that we receive, we cannot protect or control what the company does with the information collected with their device or app. You can say decline to use these devices or apps and still be in other parts of the RECOVER study.

If needed, we can try to arrange to do the study tests in your home. If you are having a home visit, a qualified health care professional from the study team, such as a nurse, will visit you at your home to perform the tests that are related to this study.

Level 2 Tests

About 1 in 3 of the people completing Level 1 tests will be asked to do Level 2 tests. Those with certain symptoms or findings in Level1 tests will have Level 2 tests. We will also choose some people without symptoms randomly (like by flipping a coin) to do Level 2 tests. You may be asked to complete these tests up to 4 times over the course of the study, but not more often than once a year.

If you are not able to do these tests in person at the study office, we may be able to arrange to have the tests done through home visits by a qualified health care professional from the study team, or at doctor's offices or laboratories near you.

Level 2 tests may include the following:

Blood tests	Blood tests are done to follow up on findings in Level 1.
Oral glucose tolerance test (OGTT)	This test measures your blood sugar (glucose) and how your body uses glucose. You will give blood samples at different times before and after you drink a sugary liquid. If you are asked to do this test, at most we will draw about 2 (30 mL) tablespoons of blood, and up to 8 (120 mL) tablespoons over 4 years.
Home sleep study	The tests measures how you sleep using a monitor. This test is done at home.
Examinations	These include testing your feeling in your legs or artery function; examining your nose, throat and ears; testing your vision (how well you can see); checking your sense of smell; or testing how well you can do basic activities like walking or using your hands.
Electrocardiogram (ECG):	This test records how your heart is beating.
Cognitive (thinking) tests	This tests your memory, attention, mood, and thinking. This takes about 20 minutes.

Approved For Period: 12/15/2022 - 8/22/2023

Pulmonary (lung) function tests	This test measures how well your lungs are working. You will breathe through a plastic tube in your mouth in different ways, such as fast, slow, deep, or shallow. You may be asked to take a medicine called albuterol to help you breathe better during this test. Albuterol is a medicine used to treat lung diseases, such as asthma. It relaxes muscles in your airways and it is taken through an inhaler.
Ultrasounds	These tests use sound waves to get a picture of your heart, kidneys or liver.
CT scan of lungs	CT scans use X rays to get a picture of your lungs. You cannot have this test if you are pregnant.

Level 3 Tests

About 1 in 5 people will be asked to do some level 3 tests. You would only do each Level 3 test once. Below is a list of low risk Level 3 tests. You will be asked to sign a separate consent form that describes the procedures and risks for any Level 3 test that has more than very low (minimal) risk.

Low risk Level 3 tests may include the following:

Blood tests	Blood tests are done to follow up on findings in Level 1 or Level 2.
Sleep study	This test measures how you sleep using a monitor. This test is done overnight at the hospital.
Examinations	These include testing your hearing and testing how your blood vessels work with a sensor on your finger
Cognitive (thinking) and mood tests	Level 3 has more detailed tests of memory, attention, mood and thinking than Level 2. These may take 1-2 hours.
Eye exam	This includes tests of your vision, eye pressure, and movement. We would use drops to dilate your eyes.
Exercise testing	While you exercise on a treadmill or bike, we will monitor your heart and breathing through a tube in your mouth. You can not have this test if you are pregnant.

Communicating with the Research Team by Text

The research team will contact you by phone, email or text messages, depending on your preference. There is no way to protect (“encrypt”) information in the messages sent by text. This means that information you send or receive by text message could be looked at by someone who was not supposed to see it, or by your mobile/cell phone provider or company. Therefore, when text messages are sent, there may be risks related to your privacy. We would like to use text messages to remind you about visits, send you links to surveys and give you other information about the study. We will not send you test results by text. Please indicate whether you agree to receive text messages from the research team:

Approved For Period: 12/15/2022 - 8/22/2023

_______ Yes, I agree to receive texts from the research team.
Initial here

_______ No, I do not agree to receive texts from the research team.
Initial here

Please make sure to keep the research team updated if your mobile/cell phone number changes during the study.

Communicating with the Research Team by Email

We may contact you by email. When we send email messages that include information about your health, like test results, we will help keep your personal information confidential by "encrypting" the message. When you receive an email with this added protection, you may have to create a username and password and log into a website to see the information.

Please make sure to keep the research team updated if your email address changes during the study.

Future Use of Survey and Test Results Data

Information that identifies you, such as your name and address, will be removed from the questionnaires and test results. After this, information may be used for future research studies or shared with other researchers without a risk of loss of confidentiality to you and we will not request additional informed consent from you to use these specimens.

Optional Future Use of Blood, Tears, Saliva, Stool and Other Samples from Your Body (Biospecimens)

With your permission, biospecimens (blood, tears, spit (saliva), poop (stool) and pee (urine)) will be sent to a storage place called the RECOVER Research Biorepository at Mayo Clinic, MN to be stored for future research tests.

Blood, tears, saliva, urine, stool and other samples in this storage place will be used mostly for research on COVID and the long-term effects of COVID, but they may also be used for research on other health problems.

One special type of future research is genetic research on the DNA in your samples. DNA is the material that makes up your genes. All living things are made of cells. Genes are the part of cells that have the instructions which tell our bodies how to grow and function. Genes are passed from parent to child.

We may also perform a whole genome analysis on your DNA sample. Usually researchers study just a few areas of your genetic code that are linked to a disease or condition. In whole genome studies, all or most of your genes are analyzed and used by researchers to study links to Long COVID.

Your study information and study samples may be shared with researchers around the world, including those with commercial interests. However, leaders of this RECOVER study control who can get your information and study samples. To use your information and study samples, researchers must ask RECOVER leaders if samples can be shared. Your name or other identifying information will not be provided to other researchers. Samples that are stored will be given a code number and only the researchers at the place you signed up to be part of this research (your study site) and the researchers who are in charge of keeping study information will have the key that links, or connects, the code number given to your study samples with your personal information. Researchers must also agree to not try to figure out who you are. If you change your mind about sharing your samples, you can withdraw your samples from the Biorepository by contacting the Study Leader named at the top of this form.

At the end of the study, your personal information will be completely removed from the RECOVER database, and there will no longer be a key to link your information with the study samples. After taking away the key, the data and study samples you provided may be used for future research studies without your consent because they are no longer linked to you. Note that after the linking key is removed, you will no longer be able to request to take back your study samples.

Please initial the line below to let us know if you want to allow your samples to be used in future research.

_______ Yes, I agree to allow my samples to be used for future research, including research on my genes.
Initial here

_______Yes, I agree to allow my samples to be used for future research, but **NOT** research on my genes.
Initial here

_______ No, I do not agree to allow my samples to be used for future research.
Initial here

5. What are the possible risks or discomforts?

Being in this study may involve some risks or discomforts from study procedures. In addition to the risks listed below, there may be risks that have not been seen before. You should contact the Study Site Leader if you are concerned about anything while you are a part of the study.

Possible loss of privacy or confidentiality
When we share your information and study samples, there is a small risk that people may get to see it who are not supposed to. Researchers will do their best to protect your privacy by keeping identifying information about who you are in a different place from the other information you are giving us and any results. We will keep your study information and study samples in as securely as possible. Researchers using your information and study samples must agree not to try to find out who you are. However, there is a small chance they may be able to find out who you are.

The research team may communicate with you electronically, such as over email or text message. While the researchers will take steps to protect your privacy and confidentiality, text messages are not encrypted and not a secure mode of communication, and there is a risk that people may see the messages who are not supposed to. The researchers will only send limited information in these messages.

Approved For Period: 12/15/2022 - 8/22/2023

When you use apps or software made by companies, such as wearable fitness trackers, wearable sleep monitors, or other mobile or web apps, there is a small risk that people outside the research study may get to see your information who are not supposed to.

Risk of surveys, office tests and ultrasounds, including:

Surveys and tests of thinking
Physical examinations
Tests of smelling, vision, hearing and sleep
Blood tests
Ultrasounds

Answering the survey questions can take a long time. This may make you tired, uncomfortable, or frustrated. You can stop or take a break if you need to. The other tests listed are routine medical procedures with minimal risk.

Risk of COVID nasal swab (COVID test using liquid from your nose)

The nasal swab test may be uncomfortable and may cause a small number of people to gag, cough or have a nose bleed.

Risk of having blood taken

Blood will be drawn through a needle placed into a vein in your arm. Having blood taken may be uncomfortable or make you feel dizzy or faint (pass out). Tell the staff right away if you feel like you might pass out. Redness, pain, bruising, bleeding, or infection may also happen where the needle goes into the skin during blood collection.

Tests of how well you see (vision testing)

There is a very low (minimal) risk from vision tests. The eye drops used to dilate your eyes (open the pupils to see in the eye) may sting. You may have glare and blurry vision for several hours while your eyes are dilated. Some people are allergic to eye drops. Some people have an increase in eye pressure which would make your eye red or painful that goes away with time. These problems will be treated if they occur.

6 minute walk test

You may get tired, lightheaded or dizzy during this test. There is minimal medical risk from this test.

Electrocardiogram (ECG)

There is minimal medical risk from ECG. The sticky pads (electrodes) that are put on your chest can sometimes cause discomfort such as redness or itching. We may need to shave your chest before we put the pads on. Shaving may also bother the skin.

Exercise testing

The main risks of exercise testing are abnormal heart beats (arrhythmias), change in blood pressure (too high or too low), heart attack (myocardial infarction), muscle, bone, or joint injury, or death. However, these problems are very rare: they happen in less than 1 in every 5,000 to 10,000 tests, with death estimated at 1 in every 20,000 tests. You can stop the test at any time if you do not feel well. You can not have this test if you are pregnant.

Radiation Exposure from CT scan

Approved For Period: 12/15/2022 - 8/22/2023

Your participation in this study may involve exposure to radiation from CT scans. You may receive a chest CT in your first year and no more than one chest CT each subsequent year for up to four years. This exposure is not necessary for your medical care, is for research purposes only and is necessary to obtain the desired research information.

Radiation has been shown to cause cancer from exposures that are significantly higher than the additional radiation dose you will receive by participating in this study. According to the International Commission on Radiological Protection (ICRP), the increased risk of health effects, such as cancer, from radiation doses of this amount is either too small to be observed or nonexistent. The effective radiation dose you will receive in your first year from these research scans is approximately 6 mSv. The total effective dose you will receive from all combined x-ray and CT scans is approximately 22 mSv. The organ receiving the highest dose in this study is the thymus. The effective dose you will receive from your participation is comparable to 7 times the yearly dose from natural environmental radiation in the US (3.1 mSv) and within the limits of 50 mSV, which is set by the FDA for individuals participating in basic research studies.

Please inform your study team if you have been exposed to radiation as a result of any other research studies or part of your clinical care. If you participate in future studies that involve the use of radiation, you should discuss it with the researchers performing those studies.

CT Scan Dye
Some of the CT scans in this study use dye in the veins (“contrast”). Some people are allergic to this dye; if you are, we will not do the study. For this reason, we will ask you about allergies to contrast or shellfish in advance. Sometimes, the dye will cause temporarily kidney damage, especially in people who already have kidney problems or are dehydrated (have not drunk enough water). For this reason, we will check how your kidneys are working first and will not do the test if your kidneys are not working right.

Risks of the Oral Glucose Tolerance Test
There is minimal medical risk from this test. After drinking the sugary liquid, you may have some side effects. Side effects may include feeling sick to your stomach (nausea), feeling sweaty, or feeling dizzy or like you might faint. People with diabetes may have high blood sugar after the test. You may need to take a dose of your regular diabetes medicine after the test if your blood sugar is high.

Risks of Pulmonary Function Test
Some people may feel dizzy or lightheaded during the test. If you are given the medicine albuterol during this test, you may have side effects. Side effects of albuterol include feeling nervous, shakiness, headache, sore throat or nostrils, and muscle aches. More serious, but less common, side effects of albuterol include a fast heartbeat or feeling like your heart is pounding.

Risks of Genetic Research
It is possible that during the study, we may find out things about your genes. You can decide whether you want to be told about these things. There can be a risk in knowing these results. For example, in the future, researchers may look at the full set of genes in your body, including looking at the exact order of DNA. This is called whole genome sequencing. New information may be found that show that you have genes that make it more likely that you will have certain health problems. Knowing this information can be stressful, lead to worry and affect your relationship with your family. There may also be benefits in knowing about genetic risks of disease; sometimes you can change your habits or have screening tests that will help you avoid disease or treat a disease earlier.

Approved For Period: 12/15/2022 - 8/22/2023

Group Risks
Although we will not give researchers your name, we will give them basic information such as your race, ethnic group, and sex. This information helps researchers learn whether the factors that lead to health problems are the same in different groups of people. It is possible that such findings could help people of the same race, ethnic group, or sex as you. However, they could also be used to support harmful stereotypes, stigmatize, or discriminate against members of a socially defined group such as race or ethnicity.

Pregnancy
Some of the tests in this form can not be performed on people who are pregnant. People of child bearing potential must have a negative pregnancy test before they can have those tests. It is important to understand that a negative pregnancy test may occur even if you are pregnant depending on the timing of the test relative to ovulation.

PRIVACY LANGUAGE FOR APPS
This study will use a mobile application to gather information for the researchers as part of this study. This mobile application is provided by CareEvolution and there are terms of use that the vendor requires of all users. You will need to review the app maker's terms of use and privacy policy, which we will provide to you. The maker of the app may retain some of the data collected through the mobile application even after the study ends. If you do not want this data collection to continue by the maker of the app after the study ends, you will need to de- install/discontinue use of the mobile application. The research team can help explain how to do this. Any data that is stored by the app or sent to the app maker is not under the control of this study, which means that the study cannot edit or otherwise delete it.

6. Can I be in the study if I am pregnant or breastfeeding?

You can be in the study if you are pregnant or breastfeeding, but you will not get any of the procedures that pose risk to the fetus or nursing baby.

7. What if new information becomes available?

During this study, we may find information that could be important to you. This includes information that might cause you to change your mind about being in the study. We will let you know as soon as possible if this kind of information is found.

You will be able to see the results of the tests done by a certified clinical laboratory in your health record. At some places, your main doctor, and other people who care for you and who can look at your health records will be able to see this information. Also, each of the clinical laboratory tests and scans done as part of this study will be looked at by an expert, who may find something that is not normal. If something that could affect your health is found, someone from the study team will talk to you in person or by phone about this new information.

Some tests may be done in a research laboratory. A research laboratory does tests where the results are just used for research. The results may not be reliable for use in your health care, or we may not know what the results mean for your health. Research laboratory tests may be done while you are in the study, or in the future after the study is finished. The law does not let us give you results of tests done in research laboratories because they are not meant for patient care.

Findings from Genetic Research

If you agreed to future research on your genes, you may choose to be told about any results we find about your genes that increase your risk of a health condition.

If you choose to be told about these results, a study doctor will speak to you in person or on the telephone to explain the information. It is important for you to know that the results may be part of your health record, which means that anybody who is allowed to see your health record (for example, your main doctor) will be able to see this information. Based on the genetic results, your health care provider may order more tests or treatments that are not part of the study, and may ask you to meet with a specially trained counselor who is expert at talking to people about the results (genetic counselor). Those tests or counseling are not part of the study, so you and/or your health insurance might have to pay for those costs.

What you decide does not change whether you can be part of the study, and does not mean that you have to have any more tests.

Please initial next to your choice below:

_______ Yes, I would like to be told about any results from looking at my genes.
Initial here

_______ No, I would not like to be told about any results from looking at my genes.
Initial here

_______ I did not agree to future research on my genes.
Initial here

8. What are the possible good things (benefits) from being part of the study?

Being part of the study may help you and your doctor better understand problems that are due to COVID. The results of the study will be important in helping patients, caregivers, and parents understand how COVID affects the body’s long-term reaction to COVID.

9. What other choices do I have if I am not part of the study?

This study does not provide treatment and the tests are being done for research purposes, not to provide you with health care. You will have to seek treatment for COVID symptoms apart from being in this study. If you do not take part in this study, you can get any tests you need for COVID care from your own health care providers. Deciding not to be part of the study will not affect your health care now or in the future, how you pay for health care or if you can get health insurance.

10. Will I be paid for being in this study?

We will pay you back for travel costs to and from the study site and any hotel costs related to the study. In order to be paid, you must give the receipts to the study staff.

Approved For Period: 12/15/2022 - 8/22/2023

Because you will be getting money to be part of this research study, you will need to give the study staff either your Social Security number or your Alien Registration number. You will also be asked to fill out a form called the IRS W9. This is because [study site] is required by laws of the United States to report to the Internal Revenue Service (IRS) any amounts that are paid to research participants that are equal to or greater than $600. You may need to pay taxes on payments for research that are greater than $600. If you do not have either of these numbers or are not willing to fill out the IRS W9 form, you may be in the study but will not get any money.

You must keep a record of all the money given to you for being part of this research study and any other research study for each year (from January to December) that you are part of. You must let us know right away when the total amount of money you get for being part of research studies is the same as or likely to be greater than $600 total (not including money to pay for the cost of travel) in any one year (from January to December).

The use of your information and study samples may lead to new tests or drugs, or other things that may be sold to make money. A patent or license may be gotten for these things to keep other people from making, using, or selling these things. There are no plans to give any money to you if this was to happen.

11. Will I have to pay for anything?

There are no costs to you related to being part of the study. The NIH will cover the costs of your being part of the study. You or your insurance company will not be asked to pay for the costs of your visits related to the study. You and/or your health insurance may be billed for the costs of health care during this study if these costs would have happened even if you were not in the study. If your insurance does not cover these costs or you do not have insurance, these costs will be up to you to take care of.

12. What happens if I am injured from being in the study?

For emergencies, call 911. If you think you have been hurt because of being part of this research study, tell the Study Site Leader as soon as you can. The Study Site Leader’s name and phone number are listed at the top of page 1 of this consent form.

If you are hurt because of being part of this research, we will put you in contact with a doctor to give you treatment if you want. We may ask your insurance company, or someone else, if appropriate, to pay for the costs of the treatment due to your being hurt, but you may also need to pay for some of this cost.

There are no plans for the [study site] or NYU Grossman School of Medicine or NYU Langone Health to pay you or give you anything else for being hurt. You do not give up the rights you have under the law by signing this form.

13. When is the study over? Can I leave the study before it ends?

This study will last for 4 years. This study may be stopped early. It is also possible that you may be taken out of the study early for the following reasons:

- The researchers in charge of the study feel it is important to remove you for your health or safety.
- You have not followed study instructions.

- The group funding the study, the main researchers in charge, or people monitoring the safety of the study decide to stop the study.
- More information about Long COVID is known so that the study is no longer needed.

If you do not wish to keep being part of the study, you are free to leave the study at any time. Leaving the study will not affect your care, how your health care is paid for, or what kind of health insurance you can get.

14. How will you protect my confidentiality (privacy)?

Your medical information is protected health information, or "PHI", and is protected by federal and state laws, such as the Health Insurance Portability and Accountability Act, or HIPAA. This includes information in your research record as well as information in your medical record at NYU Langone Health. In compliance with NYU Langone Health policies and procedures and with HIPAA, only those individuals with a job purpose can access this information.

Medical information created by this research study may become part of your medical record. We may include your research information in your medical record for several reasons, including for the billing of services provided in connection with the study, to securely document any medical services you receive, and so that other members of the NYU Langone Health community who may treat you have access to important information about your health.

You have a right to access information in your medical record. In some cases, when necessary to protect the integrity of the research, you will not be allowed to see or copy certain information relating to the study while the study is in progress, but you will have the right to see and copy the information once the study is over in accordance with NYU Langone Health policies and applicable law.

Certificate of Confidentiality
To help us further protect your confidentiality, this research is covered by a Certificate of Confidentiality from the National Institutes of Health (NIH). The NIH has issued a Certificate of Confidentiality for this research. This adds special protection for the research information (data, documents, or biospecimens) that may identify you.

Research information protected by this Certificate of Confidentiality cannot be disclosed to anyone else who is not connected with the research, without your consent. With this Certificate of Confidentiality, the researchers may not disclose or use research information that may identify you in any federal, state, or local civil, criminal, administrative, legislative, or other action, suit, or proceeding, or be used as evidence, for example, if there is a court subpoena, without your consent. However, disclosure, without your consent, is still necessary if there is a federal, state, or local law that requires disclosure (such as to report child abuse or communicable diseases).
The Certificate of Confidentiality cannot be used to refuse a request for information from appropriate government agencies responsible for project oversight.

The Certificate of Confidentiality does not prevent you from releasing information about yourself and your involvement in this research, including for your medical treatment. Federal regulations may also allow for the use or sharing of information for other scientific research.

Approved For Period: 12/15/2022 - 8/22/2023

By agreeing to be in this research and signing below, you are giving your consent to share research information with others at [study site]. This means that your research information, including lab results, x-rays, MRIs, may be included in your [study site] electronic medical record.

15. HIPAA Authorization

As noted in the Confidentiality part of this consent form above, federal laws in the U.S. have been made so that we, and the researchers working with us, health care providers, and the people who care for you protect the privacy of information that identifies you (used to know who you are) and relates to your past, present, and future physical and mental health problems. We are asking for your permission (authorization) to use and share your health information with others related to this study, in other words, in order for this research to happen, including doing and watching over the study.

Your treatment outside of this study, payment for your health care, and your health care benefits will not be affected even if you do not give permission (authorize) the use and sharing of your information for this study.

What information may be used or shared with others related to this study?

All information in your research record for this study may be used and shared with those people who are in the list at the end of this part of the consent form. Also, information in your health record that the research team believes may be important to the study may be looked at by those in the list. This includes, for example, results from your study visits, laboratory tests, body pictures, scans, other tests, surveys, and diaries.

Who may use and share information in connection with this study?
The following individuals may use, share, or get your information for this research study:

- The research team, including the Study Site Leader and other people helping with the study or who are in charge of watching over the study at [study site]
- The researchers at NYU Grossman School of Medicine, who are in charge of helping with and watching over the study at all the places across the country where the study is happening
- Non-research staff who need this information to do their jobs (such as for treatment, payment (billing), or health care operations)
- The researchers at Massachusetts General Hospital, who are in charge of storing the information for this study, the researchers at Mayo Clinic who are in charge of the research biorepository, and other RECOVER study centers or national centers in charge of storing research information
- The group that funded the study: National Institutes of Health (National Heart Lung and Blood Institute)
- The ethical review board (also called institutional review board or IRB) that oversees the research and the research quality improvement programs.
- The group that is watching over the safety of patients and families in the study (called the observational study safety monitoring board). The National Institutes of Health decides who will be in this group.
- National data repositories such as the National Center for Biotechnology Information or dbGAP

- A company hired to oversee the quality of the RECOVER research information ((Biomedical Research Alliance of New York)
- People or groups that we hire to do work for the study, such as data storage companies, insurers, and lawyers
- Governmental agencies in charge of watching over or overseeing the research (for example, the US Department of Health and Human Services).
- Health care providers, including your doctors and others who care for you related to this study, and laboratories or other people who are looking at your health information as part of this study.
- Other places that are involved in this research

Your information may be re-disclosed (shared) or used for other reasons if the person who gets your information is not required by law to protect the privacy of the information.

What if I do not want to give permission to use and share my information for this study?
Signing this form is voluntary. You do not have to give us permission to use and share your information, but if you do not, you will not be able to be part of this study.

Can I change my mind and withdraw (take back) permission to use or share my information?
Yes, you may withdraw or take back your permission to use and share your health information at any time for this research study. If you withdraw (take back) your permission, we will not be able to take back information that has already been used or shared with others. To withdraw (take back) your permission, you must write by mail or by email to the Study Site Leader or the Study Leader for the study shown at the top of page 1 of this form. If you withdraw (take back) your permission, you will not be able to stay in this study.

How long may my information be used or shared?
Your permission to use or share your personal health information for this study will never end unless you withdraw it (take it back).

16. The Institutional Review Board (IRB) and how it protects you

The Institutional Review Board (IRB) reviews all research involving people before it can be started and then as long as the study continues. The primary concern of the IRB is for the protection of the people participating in the study. For questions about your rights while participating in the study contact the NYU IRB Office number on (212) 263-4110. The NYU School of Medicine's IRB is made up of doctors, nurses, scientists, and people from the community.

17. Who can I call with questions, or if I'm concerned about my rights as a research participant?

You can call the Institutional Review Board (IRB) with your questions or concerns. Our telephone numbers are listed below. Ask questions as often as you want. Stuart Katz, MD is the person in charge of this research study. His name and phone number are listed on the first page of this form. If you want to speak with someone not directly involved in this research study, please contact [insert name of contact or IRB]. You can call them at [insert contact information].

- You can talk to them about:
- Your rights as a research participant

Approved For Period: 12/15/2022 - 8/22/2023

- Your concerns about the research
- A complaint about the research. Also, if you feel pressured to take part in this research study, or to continue with it, they want to know and can help.

A description of this clinical study will be available on http://www.ClinicalTrials.gov. This website will not include information that can identify you. At most, the website will include a summary of the results. You can search this website at any time.

When you sign this form, you are agreeing to take part in this research study as described to you. This means that you have read the consent form, your questions have been answered, and you have decided to volunteer.

Name of Participant (Print)	Signature of Participant	Date

Name of Person Obtaining Consent (Print)	Signature of Person Obtaining Consent	Date

Witness to Consent Process for Non-English-Speaking Participants (using a translated consent form OR "Short Form" in Participant's Spoken Language)

Statement of Witness
As someone who understands both English and the language spoken by the participant, I represent that the English version of the consent form was presented orally to the participant in the participant's language and that the participant was allowed to ask questions.

Name of Witness (Print)	Signature of Witness	Date

Witness to Consent of a Participant Who Cannot Read or Write

Statement of Witness
I represent that the consent form was presented orally to the participant in the participant's language, that the participant was allowed to ask questions, and that the participant has indicated his/her consent and authorization for participation by (check box that applies).

- [ ] Participant making his/her own "X" above in the participant signature line
- [ ] Participant showed approval for participation in another way; describe:

Name of Witness (Print)	Signature of Witness	Date

Approved For Period: 12/15/2022 - 8/22/2023
