# Supplementary material for: Researching COVID to Enhance Recovery (RECOVER) adult study protocol: Rationale, objectives, and design
Source: PLoS One. 2023 Jun 23;18(6):e0286297. doi: 10.1371/journal.pone.0286297 (PMC10289397; doi:10.1371/journal.pone.0286297)
Supplement: Attachment — Submitted filename: Response to Reviewers.docx [file pone.0286297.s014.docx]

**Editor comments**

1. Please ensure that your manuscript meets PLOS ONE's style requirements, including those for file naming. The PLOS ONE style templates can be found at <https://journals.plos.org/plosone/s/file?id=wjVg/PLOSOne_formatting_sample_main_body.pdf> and 
<https://journals.plos.org/plosone/s/file?id=ba62/PLOSOne_formatting_sample_title_authors_affiliations.pdf>

**Response:** Thank you; we confirm that the paper is now formatted according to PLOS ONE’s style requirements. We have revised file names as appropriate.

2. We note that you have indicated that data from this study are available upon request. PLOS only allows data to be available upon request if there are legal or ethical restrictions on sharing data publicly. For more information on unacceptable data access restrictions, please see <http://journals.plos.org/plosone/s/data-availability#loc-unacceptable-data-access-restrictions>.

In your revised cover letter, please address the following prompts:

a) If there are ethical or legal restrictions on sharing a de-identified data set, please explain them in detail (e.g., data contain potentially sensitive information, data are owned by a third-party organization, etc.) and who has imposed them (e.g., an ethics committee). Please also provide contact information for a data access committee, ethics committee, or other institutional body to which data requests may be sent.

**Response:** RECOVER follows the NIH Policy for Data Management and Sharing NOT-OD-21-013 ([https://grants.nih.gov/grants/guide/notice-files/NOT-OD-21-013.html](https://urldefense.com/v3/__https:/secure-web.cisco.com/1KfBgtNE-Sw036pGTB2P9ZfpE8dD8T7AEbgL0RlIfK2OVSVUV4QSEX-5Jzjvm3EjD8aFKrGIoMWWHd1SaJnZ45U3cv6ELaV6RkI5iUGmQz49ANepoH9oV9DsmN0U6ArGIh8vT452R1kN-IAljhFajwCy1nw3HH0fY2zFxtkNZxfErzePVUBduAW_RMaUdyVqRPegzt_9B_6zZZ2u2gHjOheVKmnp3RX10ij0M5uohwHzRyOvzIgXqoUHAUAXtPZHnvzdaIZpwSWrijYfQVYypso0cg2gvOUIh5tRDw-tCOoenamC3Y49nvZgHKfDJ4CtH/https*3A*2F*2Fgrants.nih.gov*2Fgrants*2Fguide*2Fnotice-files*2FNOT-OD-21-013.html__;JSUlJSUlJQ!!MXfaZl3l!e_83afqbGVz3tZkWIvS8rw80DFDK9OV5H41w6ibnw_FKGMskytY6yOf2f5cuqCR9ag_4rx3FOeATig5vAutWlCr19O8$)) which stipulates that “researchers will maximize the appropriate sharing of scientific data, acknowledging certain factors (i.e., legal, ethical, or technical) that may affect the extent to which scientific data are preserved and shared” and strongly encourages the use of established repositories for this purpose. Accordingly, once the study is complete and all data cleaned and locked, the data will be made available to researchers through the NIH public repository, BioData Catalyst, according to the policies of NIH and NHLBI. At present, however, such data are not available as we are still enrolling new subjects and conducting follow up on existing enrollees. Instructions for requesting individual-level data are available on BioData Catalyst at [https://biodatacatalyst.nhlbi.nih.gov/resources/data/](https://urldefense.com/v3/__https:/biodatacatalyst.nhlbi.nih.gov/resources/data/__;!!MXfaZl3l!aPoA73Cwz8E6SFlB5ZIEEm0D9niAlBAejMEoV_SKo8k3-wVg358LB1JP2UN7QYqTVjDpiHfkc0BPhohf74kGC1oW44M$). Researcherse may apply for data access in dbGaP. Upon approval, users may begin accessing requested data in BioData Catalyst. For questions about availability, the BioData Catalyst team can be contacted at [https://biodatacatalyst.nhlbi.nih.gov/contact](https://urldefense.com/v3/__https:/biodatacatalyst.nhlbi.nih.gov/contact__;!!MXfaZl3l!aPoA73Cwz8E6SFlB5ZIEEm0D9niAlBAejMEoV_SKo8k3-wVg358LB1JP2UN7QYqTVjDpiHfkc0BPhohf74kGmh0s0mk$).

b) If there are no restrictions, please upload the minimal anonymized data set necessary to replicate your study findings as either Supporting Information files or to a stable, public repository and provide us with the relevant URLs, DOIs, or accession numbers. For a list of acceptable repositories, please see <http://journals.plos.org/plosone/s/data-availability#loc-recommended-repositories>.

**Response:** N/A. This manuscript does not include any study findings; it is a study protocol paper.

We will update your Data Availability statement on your behalf to reflect the information you provide

3. Thank you for stating the following in the Acknowledgments Section of your manuscript:

"We would like to thank the National Community Engagement Group (NCEG), all patient, caregiver and community representatives, and all the participants enrolled in the RECOVER initiative.

Membership of the RECOVER Initiative is provided in a supplemental appendix file."

We note that you have provided funding information that is not currently declared in your Funding Statement. However, funding information should not appear in the Acknowledgments section or other areas of your manuscript. We will only publish funding information present in the Funding Statement section of the online submission form. 
Please remove any funding-related text from the manuscript and let us know how you would like to update your Funding Statement. Currently, your Funding Statement reads as follows:

"National Institutes of Health (NIH) Agreement OTA OT2HL161847 (SDK, LIH), OT2HL161841 (ASF), OT2HL156812 (LTN). <https://www.nih.gov/>

The funders did not and will not have a role in study design, data collection and analysis, decision to publish, or preparation of the manuscript."

Please include your amended statements within your cover letter; we will change the online submission form on your behalf

**Response:** Thank you, we have removed the Funding statement and the Competing Interest text from the manuscript. The funding statement should read: This study is funded by the National Institutes of Health (NIH) through Other Transactional Agreements OT2HL161847, OT2HL161841 and OT2HL156812 as part of the Researching COVID to Enhance Recover (RECOVER) Research Initiative. No funding source had any role in the collection, analysis and interpretation of data; in the writing of the report; or in the decision to submit the article for publication.” We have also included this statement in the revised cover letter. Note that we have now included the list of RECOVER consortia members in the Supplementary Material as Tables S6-S8. Please also note that we inadvertently left one author off in the initial submission and have now added him back in (PJ Utz).

Please review your reference list to ensure that it is complete and correct. If you have cited papers that have been retracted, please include the rationale for doing so in the manuscript text, or remove these references and replace them with relevant current references. Any changes to the reference list should be mentioned in the rebuttal letter that accompanies your revised manuscript. If you need to cite a retracted article, indicate the article’s retracted status in the References list and also include a citation and full reference for the retraction notice.

**Response:** We have carefully reviewed the reference list and confirm that it is now complete and correct. We have added one reference to the medXriv version of the RECOVER pregnancy protocol (now under review at PLOS ONE) and two statistical references.

**Reviewer Comments**

Reviewer #1: Sample size: The primary aims of this study are more focused on characterizing the prevalence, symptoms, and other factors related to PASC. Therefore, the sample size should not be based solely on comparisons between infected and non-infected individuals. Additionally, the effect sizes used in the calculation were not clinically justified. It is unclear how the targeted sample sizes were determined for subgroups in Table 2.

**Response:** Thank you for this comment. We have expanded the sample size calculation section of the manuscript to address these concerns. The revised manuscript includes three sample size calculations to address RECOVER primary aims: 1) the minimum detectable effect size for the difference in risk of PASC symptom for a) “acute” infected (enrolled within 30 days of infection) vs “acute” uninfected (enrolled within 30 days of a negative test) participants and for all (“acute” and “post-acute”) infected vs. all uninfected participants; 2) among all infected participants, the minimum detectable difference in the probability of a PASC symptom between those with and without a risk factor; and 3) among all infected participants with a PASC symptom, the minimum detectable difference in the probability of recovery among those with and without a risk factor. The sample sizes in Table 2 were selected to achieve detectable effect sizes selected by the clinician investigators as being clinically relevant corresponding to these three objectives, overall and within subgroups comprised of 25% of the participants.

The purpose and technique details of the machine learning model are not clearly described.

**Response:** We clarify in the revised manuscript that we will apply variable selection to identify combinations of symptoms that differentiate participants with and without a history of infection. Balancing weights will be applied to account for any differences between infected and uninfected participants.

It is unclear how the PASC subgroups will be analyzed.

**Response:** We added detail on the use of a multinomial model to investigate associations between risk factors and PASC subgroups. We also clarify that the competing and semi-competing risks analysis is intended to assess associations between risk factors and each PASC subgroup.

A longitudinal model may be considered for analyzing repeated measures.

**Response:** In the revised manuscript we clarify that the analysis of repeated measures will be achieved by converting these data into a time to event outcome, e.g., time to PASC recovery, and survival analyses will be implemented.

Reviewer #2: It is a well written protocol. It has ambitious but achievable objectives. The methodology is adequate. The large number of participating centers ensures the achievement of the sample size. The most important limitation is the lack of a standardized definition of long COVID-19. However, with the findings of this study a better characterization could be achieved.

**Response:** Thank you, we appreciate this comment. Indeed, a core goal of RECOVER is to generate a definition of long COVID.

While revising your submission, please upload your figure files to the Preflight Analysis and Conversion Engine (PACE) digital diagnostic tool, [https://pacev2.apexcovantage.com/](https://urldefense.com/v3/__https:/pacev2.apexcovantage.com/__;!!MXfaZl3l!cbQwDITGl25RyS0XvhwKEUp19fr4lSetLrO49q1rKQA1uiSqsZR7R3pxo6drpqLTf4mKeLqiooirxQ5Mppmx$). PACE helps ensure that figures meet PLOS requirements. To use PACE, you must first register as a user. Registration is free. Then, login and navigate to the UPLOAD tab, where you will find detailed instructions on how to use the tool. If you encounter any issues or have any questions when using PACE, please email PLOS at [figures@plos.org](mailto:figures@plos.org). Please note that Supporting Information files do not need this step.

**Response:** Thank you. Our main manuscript includes one figure. We uploaded the Figure to PACE, which converted it to a valid TIF file. We have now replaced the original Figure in the online submission system with the PACE corrected figure.
